# CRISPR-Cas target recognition for sensing viral and cancer biomarkers

**DOI:** 10.1093/nar/gkae736

**Published:** 2024-08-27

**Authors:** Shadi Rahimi, Sri Renukadevi Balusamy, Haribalan Perumalsamy, Anders Ståhlberg, Ivan Mijakovic

**Affiliations:** Division of Systems and Synthetic Biology, Department of Life Sciences, Chalmers University of Technology, SE-41296 Gothenburg, Sweden; Department of Food Science and Biotechnology, Sejong University, Gwangjin-gu, Seoul, Republic of Korea; Center for Creative Convergence Education, Hanyang University, Seoul 04763, Republic of Korea; Research Institute for Convergence of Basic Science, Hanyang University, Seoul 04763, South Korea; Sahlgrenska Center for Cancer Research, Department of Laboratory Medicine, Institute of Biomedicine, Sahlgrenska Academy at University of Gothenburg, Gothenburg, Sweden; Wallenberg Centre for Molecular and Translational Medicine, University of Gothenburg, Gothenburg, Sweden; Region Västra Götaland, Department of Clinical Genetics and Genomics, Sahlgrenska University Hospital, Gothenburg, Sweden; Division of Systems and Synthetic Biology, Department of Life Sciences, Chalmers University of Technology, SE-41296 Gothenburg, Sweden; The Novo Nordisk Foundation Center for Biosustainability, Technical University of Denmark, Lyngby, Denmark

## Abstract

Nucleic acid-based diagnostics is a promising venue for detection of pathogens causing infectious diseases and mutations related to cancer. However, this type of diagnostics still faces certain challenges, and there is a need for more robust, simple and cost-effective methods. Clustered regularly interspaced short palindromic repeats (CRISPRs), the adaptive immune systems present in the prokaryotes, has recently been developed for specific detection of nucleic acids. In this review, structural and functional differences of CRISPR-Cas proteins Cas9, Cas12 and Cas13 are outlined. Thereafter, recent reports about applications of these Cas proteins for detection of viral genomes and cancer biomarkers are discussed. Further, we highlight the challenges associated with using these technologies to replace the current diagnostic approaches and outline the points that need to be considered for designing an ideal Cas-based detection system for nucleic acids.

## Graphical Abstract

**Graphical Abstract:**
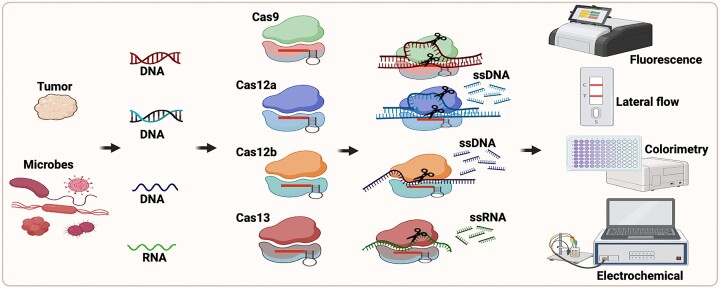

## Introduction

Nowadays most nucleic acid-based diagnostics are based on polymerase chain reaction (PCR) and sequencing approaches. Despite high sensitivity, there are shortcomings with these methods, such as complicated workflows, handling of fragile enzymes, relying on advanced instruments and skilled operators. Clustered regularly interspaced short palindromic repeats (CRISPRs) are adaptive immune systems, that are present in about half of the sequenced bacteria and almost all archaea, protecting them from viruses and other invading DNAs (1). The enzymatic capability of CRISPR–Cas systems for specific targeting of DNA sequences, insertion and deletion *in vivo* promoted numerous advancements in the field of gene therapy. However, simple, and specific recognition of nucleic acids by CRISPR-Cas can also be utilized for different diagnostic applications.

**Figure 1.**
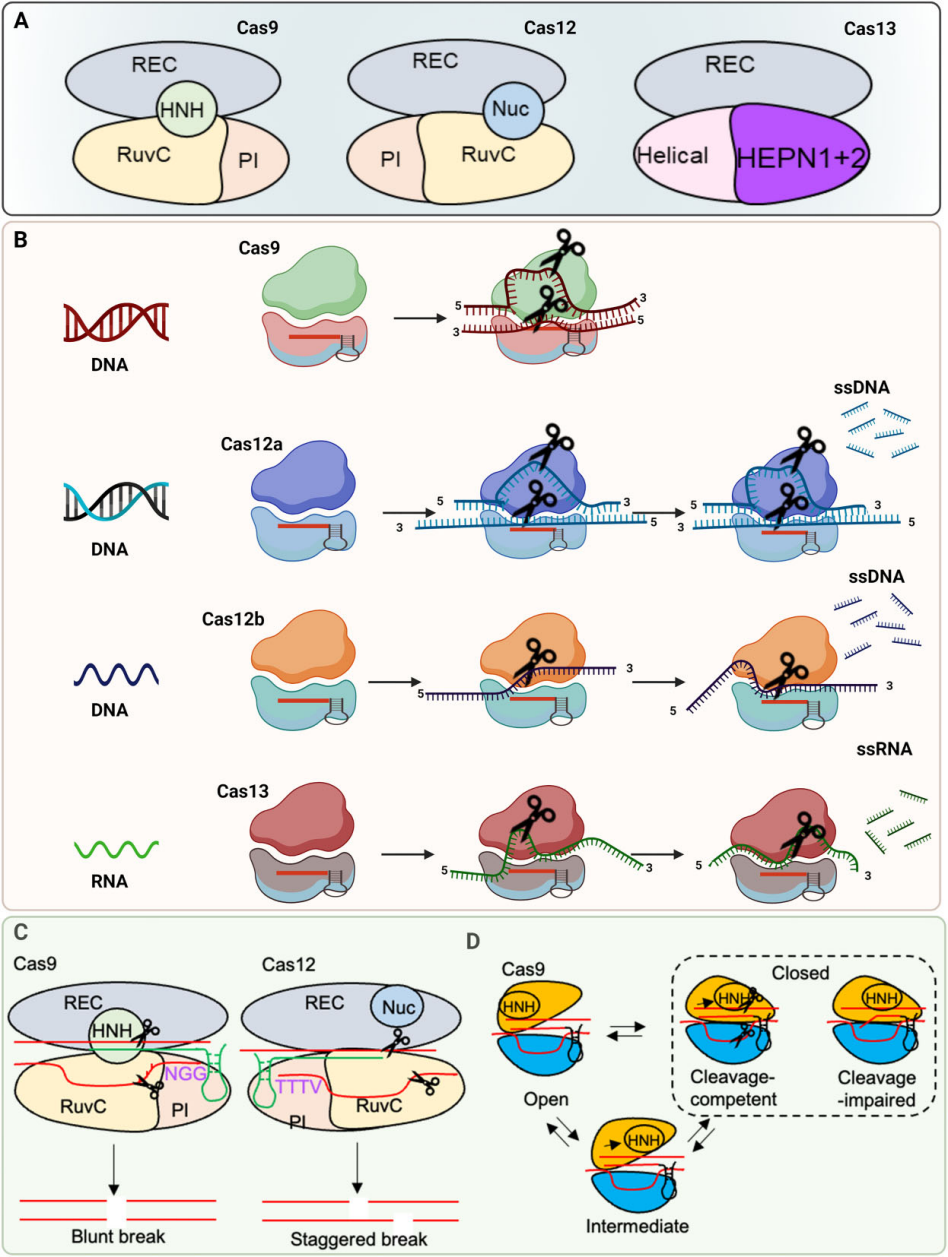
(**A**) The structure of Cas9, Cas12 and Cas13 proteins. Cas9 comprises RuvC and HNH nuclease domains, Cas12 comprises single RuvC nuclease domain, and Cas13 contains two conserved HEPN (higher eukaryotes and prokaryotes nucleotide-binding) domains that cleaves RNA. (**B**) Mechanism of function of Cas9, Cas12 and Cas13 proteins on DNA and RNA. While Cas9 and Cas12 enzymes target DNA, Cas13 cleaves single-stranded RNA targets. Cleavage of surrounding nontargeted nucleic acids after recognition of target is called collateral cleavage activity. Collateral cleavage activity of Cas12 is against ssDNAs while that of Cas13 is against ssRNAs. (**C**) Cas9 and Cas12 cleavage of target. For Cas9, the PAM sequence (5′-NGG-3′) is located downstream of the spacer on the non-template strand and recognized by the PI domain. The HNH and RuvC domains in Cas9 cleave template and non-template at 3 base pairs upstream of the PAM, respectively, thereby generating a blunt double strand break (DSB). For Cas12, the PAM, typically 5′-TTTV-3′, is located upstream of the spacer and a single nuclease RuvC cut DNA strands in the same nuclease site, generating the staggered double strand break (DSB). (**D**) Cas9 fluctuates among three major conformational states including open, intermediate, and closed states, that reflects conformational mobility of the catalytic HNH domain.

Based on the architecture of CRISPR array and the signature interference effector, these systems are divided into two major classes, six types (I to VI) and 33 sub-types (2). Class 1 systems are composed of multi-subunit protein complexes, whereas class 2 systems are composed of a single-effector multidomain protein. Simpler architecture makes class 2 systems the preferred candidates for genome-editing approaches. Most commonly used class 2 CRISPR type II and type V systems for biosensing include the single-component effector protein Cas9 (comprising RuvC and HNH nuclease domains), and Cas12a (previously named Cpf1, with a single RuvC nuclease domain) (Figure 1A). These CRISPR-Cas systems provide timely diagnosis of viral infection using both DNA and RNA genomes and therefore can be used in versatile point-of-care (POC) diagnostic applications (3).

The design principles for CRISPR RNAs for different effector proteins used in CRISPR diagnostics have been previously reviewed (4). Basis for the action of these RNA-guided nucleases, such as Cas9 is that the CRISPR RNA (crRNA) recognizes foreign DNA (dsDNA), forms an R-loop structure using ∼20 nts of the crRNA with the target strand of the target DNA, displaces the non-target strand for DNA interference, and cleaves target DNA (5,6). However, there are distinct evolutionary origins for Cas9 and Cas12a and they display different structure, and hence distinct molecular mechanisms of target DNA binding and cleavage.

While the Cas9 and Cas12 enzymes target the DNA, Abudayyeh *et al.*, (2016) characterized the class 2 type VI CRISPR-Cas effector Cas13, previously known as C2c2, guided by a single crRNA that can cleave single-stranded RNA targets (Figure 1B) (7). Interestingly, the Cas13 enzyme was first realized as a potential diagnostic tool (8). East-Seletsky *et al.* (8) discovered that the RNA-guided trans-endonuclease activity of Cas13 could be used to cleave a fluorophore quencher-labelled reporter RNA, leading to an increased fluorescence upon target-RNA-triggered RNase activation.

In this review, we discuss most of the recent findings in this area, classified from a specific perspective, i.e. based on the type of Cas protein and their applications in detection of viral and cancer biomarkers. We discuss the challenges associated with using CRISPR-Cas in diagnostics and we also outline how these detection platforms can be improved.

## CRISPR-Cas function

### Cas9

Cas9 is a multi-domain DNA endonuclease[6], and it is guided by a dual trans-activating CRISPR RNA (tracrRNA): crRNA or a chimeric single guide RNA (sgRNA). Cas9 makes a bilobed structure when it is in complex with the respective RNAs. The RNP complex formation used for recognition and degradation of target DNA. The respective RNA is complementary to a 20-base pair (bp) sequence in a DNA molecule, which is a target for a site-specific double-stranded DNA break caused by CRISPR-Cas9 (9). First, Cas9 needs to recognize a protospacer-adjacent motif (PAM) on the DNA sequence. PAM is a short DNA sequence (usually 2–6 bp in length) that follows the DNA region targeted for cleavage by the CRISPR system. For DNA targeting by *Streptococcus pyogenes* Cas9, the PAM sequence is located downstream of the target DNA on the non-template strand and recognized by the PAM interacting (PI) domain that is primed for recognizing 5′-NGG-3′ PAM. It is notable that the PAM sequence would be different for Cas9 from different sources for example the PAM sequence for *Neisseria meningitidis* Cas9 is NNNNGATT (10). Then, complementary pairing between the sgRNA and the target DNA leads to the formation of an RNA–DNA heteroduplex in the PAM-proximal region. After hybridization of the DNA and RNA, the pairing extends to the distal region and positions the Cas9 active site adjacent to 3 bp upstream of the PAM, which is where the cleave of the target DNA occurs (11). Finally, the catalytic sites of HNH and RuvC domains in Cas9 cleave template and non-template strands at 3 bp upstream of the PAM, respectively, thereby generating a blunt double strand break (DSB) (Figure 1C). A ∼10 nucleotide (nt) sequence adjacent to the PAM determines the specificity of target DNA binding in Cas9. Cas9 can tolerate some mismatches with sgRNA, specifically when the mismatches are present at the PAM-distal end (12), which results in off-target DNA cleavage. However, specificity of Cas9 can be improved by engineering the enzyme or using truncated sgRNA (13,14).

The *S. pyogenes* Cas9 (SpCas9) has 1368 amino acids, and its conformation is dynamic during DNA cleavage. The conformational dynamics of Cas9 and interactions between Cas9 and the RNA/DNA heteroduplex govern its nuclease activity and target specificity. Cas9 adopts different forms while interacting with RNA/DNA, including apo (unbound), sgRNA-bound and double-stranded DNA (dsDNA)/sgRNA-bound. Based on the interaction with the target DNA, the Cas9 fluctuates among three major conformational states including open, intermediate, and closed states while turning to different forms, that mainly reflects significant conformational mobility of the catalytic HNH domain (Figure 1D).

### Cas12a

There are some functional similarities between Cas12a and Cas9, despite their distinct evolutionary origins. *Francisella novicida* Cas12a (FnCas12a) is somewhat smaller than Cas9, with 1307 amino acids (15). Like Cas9, Cas12a multidomain effector protein makes a bilobed structure when it is in complex with the respective RNAs. Cas12a requires only a single RNA molecule, the crRNA. Cas12a has only one nuclease site in the RuvC domain (Figure 1A), and it also has an RNA processing site. In fact, Cas12a protein adopts distinct mechanism of RNA processing, PAM recognition, target DNA binding and eventually catalysis compared to Cas9.

For Cas12a, the PAM, typically 5′-TTTV-3′, is located upstream of the target DNA and it recognizes A-T rich sequences. After recognizing the PAM, target DNA is unzipped and hybridized with the RNA. A short sequence of ∼5–6 nt adjacent to the PAM is crucial for specificity of target DNA binding by Cas12a. Cas12a possess a single nuclease site, thereby DNA strands are cut in the same nuclease site, generating the staggered double strand break (DSB) (Figure 1C).

Once Cas12a/Cas12b-crRNA is bound with the target dsDNA/ssDNA, it can catalyze site-specific cleavage activity on target dsDNA/ssDNA (16,17). Furthermore, Cas12-crRNA bound with target dsDNA/ssDNA can trigger the nonspecific ssDNA cleavage activity (Figure 1B). However, the kinetics of Cas12-crRNA for site-specific target dsDNA/ssDNA cleavage and nonspecific ssDNA cleavage are basically different. Site-specific target dsDNA/ssDNA cleavage carries out a single turnover, whereas nonspecific cleavage performs multiple turnovers. It means that Cas12–crRNA complexed with target dsDNA/ssDNA remains bound even after site-specific cleavage, while the complex working on non-specific ssDNA releases its PAM-distal cleavage products from the RuvC active site, generating renewed access to substrate, and turnover (18). Interestingly, once Cas12a is bound with the target ssDNA, it can catalyze non-specific ssDNA cleavage at a rate of ∼3 turnovers per second, which is much slower than ∼17 turnovers per second once it is bound with the target dsDNA (16). It can be proposed that the non-target strand of target dsDNA may help Cas12a to acquire an optimal conformation for non-specific ssDNA cleavage. But an interesting point is that Cas12b differs from Cas12a in target preference for non-specific ssDNA cleavage. Cas12b–crRNA complex with the target ssDNA compared to target dsDNA showed higher non-specific ssDNA cleavage activity (17). Overall, Cas12 is structurally different from Cas9, resulting in different functions and can therefore be used for different sensing applications (Tables 1 and 2).

**Table 1.** CRISPR-Cas based viral genome detection

CRISPR methods	Sample	Microbial strains detection	Gene/Genome/ samples used	Detection methods	Clinical relevance/ sensitivity	Detection limit, operation time, cost, point of care (POC), orthologue, temperature (Tm) control, single base resolution	Reference
Cas13	RNA	HeLa cell total RNA	Target ssRNA, β-actin	Fluorescence	–	10 pM, 120 and 180 min, LbuCas13a, LseCas13a, LshCas13a, Tm control (37°C)	(8)
Cas13 with Csm6	RNA	Dengue or Zika virus	Dengue or Zika virus single-stranded RNA, mutations in patient liquid biopsy	Fluorescence, lateral flow	Clinically relevant*	2 amol, <90 min, low cost for many targets detection, LwaCas13a and EiCsm6, Tm control (37°C), single base resolution	(39)
HUDSON + SHERLOCK, Cas13	DNA or RNA	Zika and Dengue virus	Four Dengue virus serotypes, region-specific strains of zika virus, viral single-nucleotide polymorphisms	Fluorescence, lateral flow	Clinically relevant*	1 copy/μl, <2 h, LwCas13a, nuclease inactivation 37–50°C; viral inactivation 64–95°C; Tm control (37°C for fluorescence and RT for lateral flow), single base resolution	(40)
Cas13 (CARVER)	RNA	Lymphocytic choriomeningitis virus, influenza A virus, vesicular stomatitis virus	Viral genome	Fluorescence	–	<2 h, LwaCas13a and PspCas13b, Tm control (37°C), single base resolution	(43)
Cas13	RNA	SARS-CoV-2	Nasal swab RNA	Fluorescence	Clinically relevant**	∼100 copies/μl, 30 min, Lbu Cas13a, Tm control (37°C), low cost using mobile phone, POC	(46)
SHERLOCK, Cas13	DNA or RNA	Specific strains of Zika and Dengue virus, pathogenic bacteria, genotype human DNA	Clinical isolates (serum or urine), clinical isolates of *Klebsiella pneumoniae* with two resistance genes: *Klebsiella pneumoniae* carbapenemase (KPC) and New Delhi metallo-β-lactamase 1 (NDM-1), saliva, blood	Fluorescence	Clinically relevant*	Attomolar levels, POC, LwCas13a, Tm control (37°C), single base resolution	(38)
Cas13	RNA	Ebola	Purified Ebola RNA	Fluorescence	Not clinically relevant***	∼20 pfu/ml (5.45 × 10^7^ copies/ml), 5 min, low cost with integrated fluorometer and microfluidic system, POC, LwCas13a, Tm control (37°C)	(41)
SHINE-Cas13	RNA	SARS-CoV-2	Viral RNA from unextracted samples	Fluorescence, colorimetry, lateral flow	Clinically relevant**	>1000 copy/μl, 50 min, LwCas13a, Tm control (37°C), single step	(42)
CAMEN-Cas13	RNA	169 viruses, subtyping of influenza A strains, HIV drug-resistant mutations	169 human-associated viruses, Haemagglutinin and neuraminidase subtypes H1–H16 and N1–N9 of influenza A virus, mutations in HIV reverse transcriptase coding sequence	Fluorescence	More sensitive than NGS	Attomolar, 3 h, low cost with multiplexing and throughput, LwCas13a, Tm control (37°C)	(44)
Cas13, APC-Cas	DNA	*Salmonella enteritidis*	Clone SE-3	Fluorescence	More sensitive and accurate than real-time PCR	One colony-forming unit, 140 min, low cost reagent, LbCas13a, Tm control (37°C), single cell detection	(95)
Cas12a	RNA	Ebola virus	VP30 protein of the Zaire strain of the Ebola	Fluorescence, μPAD	–	11 aM, low cost materials, LbCas12a, Tm control (37°C)	(121)
Cas12b	DNA	HPV16, human ABO blood genotyping	Synthetic HPV16 dsDNA, *ABO* genes	Fluorescence	More sensitive than Cas12a-DETECTR	Sub-attomolar, AaCas12b, Tm control (37°C), single base resolution	(87)
Cas12a, E-CRISPR	DNA	HPV16, PB19 viruses	L1-encoding gene of HPV1, ssDNA PB19	Fluorescence	–	50 pM, 30 min, low cost electrochemical biosensing, POC, LbCas12a and AsCas12a, Tm control (37°C)	(122)
Cas12a	DNA	African Swine Fever Virus (ASFV)	Porcine plasma	Fluorescence	More sensitive than enzyme-linked immunosorbent assay (ELISA) or lateral flow assay (LFA), less sensitive than PCR	1 pM, 2 h, POC, LbCas12a, Tm control (37°C)	(58)
AIOD-CRISPR, Cas12a	RNA	SARS-CoV-2	SARS-CoV-2 nucleoprotein gene COVID-19 clinical swab	Fluorescence, colorimetry	Clinically relevant**	∼5 copies, 20 min, low-cost hand warmer as incubator, POC, LbCas12a, single Tm control (37°C), single base resolution, one pot	(62)
HOLMES, Cas12a	DNA, RNA	Human SNP genotypes, DNA viruses, RNA viruses, virus strains	SNP rs1014290, pseudorabies virus, Japanese encephalitis virus, PRV Ra classical strain, cmz variant strain, Bartha-K61 vaccine strain, JEV NJ2008 strain, live-attenuated vaccine strain SA14-14-2	Fluorescence	More sensitive than PCR alone or quantitative PCR using the SYBR Green method	10 aM, 1 h, low cost with no expensive reagents and no special instruments, LbCas12a/ OsCas12a/Lb5Cas12a/ FnCas12a, Tm control (37°C), single base resolution	(59)
miSHERLOCK-Cas12a	RNA	SARS-CoV-2	Viral RNA, B.1.1.7, B.1.351, P.1 variants	Fluorescence	Clinically relevant**	1000 copies/ml, low cost with reusable heater and temperature regulator electronics, POC, LbCas12a, Tm control (37°C), one pot	(63)
CANTRIP-Cas12a	DNA	Anthrax lethal factor gene	*lef* gene	Fluorescence	Sensitivity needs improvement	10 pM, 4 h, POC, LbCas12a, isothermal (37°C)	(64)
Cas12a	RNA	Dengue fever virus	Viral RNA	Electrochemical	Clinically relevant for urine samples*	100 fM, 30 min, Cpf1 provided from IDT, single base resolution, without amplification	(65)
CRISPR-Dx/ Cas12a	DNA	HPV	HPV-16, HPV-18	Fluorescence	2 orders of magnitude sensitive than ssDNA reporter	10 fM, 2 h, Cas12a orthologs from Lachnospiraceae bacterium ND2006 and *Acidaminococcus* sp., Tm control (37°C)	(83)
Cas12a	DNA	HBV, HPV	HBV, HPV-16, HPV-18	Surface-enhanced raman scattering	More sensitive than electrochemical (123) and electrochemiluminescence (124) detection	Attomolar, 20 min, low cost with low-cost Raman instrument, Cas12a from IDT, RT	(68)
Cas12a	RNA	SARS-CoV-2	E and N genes, Orf1ab gene fragment	Colorimetry	95.12% consistency with the gold standard RT-qPCR	50 RNA copies per reaction, 50 min, POC, LbCas12a, Tm control (37°C)	(118)
Cas12a	RNA	SARS-CoV-2	N and Orf1ab gene	Colorimetry	LODlower than real-time RT-PCR (125)	1 copy of viral genome per test, 1 h, Cas12a from New England Biolabs, isothermal	(119)
Cas12a, Cas13	RNA, DNA	Transgenic rice, African swine fever virus, 16S rDNA, 16S rRNA	35S promoter, VP72 gene, miRNA-17, 16S rDNA, 16S rRNA	Colorimetry	Less sensitive than qPCR	200 copies, 1 nM, 1 h, low cost with detection of *16S rRNA*, AsCpf1, LbuCas13a, Tm control (37°C), single base resolution	(120)
Cas12a	DNA	Methicillin-resistant *Staphylococcus aureus*	*mecA* gene	Electrochemical, fluorescence	As accurate as gold-standard PCR	3.5 fM, 90 min, Cas12a from New England Biolabs, RT, single base resolution	(51)
Cas12	RNA	SARS-CoV-2	E (envelope) and N (nucleoprotein) genes of SARS-CoV-2 in respiratory swab RNA extracts	Fluorescence, lateral flow	Clinically relevant**	10 copies/μl, <40 min, POC, LbCas12a, Tm control (37°C), single base resolution	(61)
STOP, Cas12b	Viral RNA	SARS-CoV-2	Gene N encoding the SARS-CoV-2 nucleocapsid protein, swab samples from patients	Fluorescence, lateral flow	Sensitivity similar to RT-qPCR	33 copies per milliliter, less than 1 h, AacCas12b and AapCas12b, one pot in a single temperature	(60)
HOLMESv2: Cas12b	DNA or RNA	SNP, virus RNA, human cell mRNA, circular RNA, target DNA methylation degree	SNP locus (rs5028), JEV virus, mature mRNA (GAPDH) and circular RNA (CDS1as), methylation degree of M3 site in the promoter of COL1A2	Fluorescence	More sensitive than HOLMES	10–8 nM, 120 min, low cost, AacCas12b, constant temperature, single base resolution	(17)
FLASH, Cas9	DNA, RNA	Antimicrobial resistance genes	Antimicrobial resistance genes in pneumonia-causing gram-positive bacteria, drug resistance in the malaria parasite *Plasmodium falciparum*	NGS	Difficult to determine sensitivity and specificity in clinical sample	Sub-attomolar, low materials cost, *S. pyogenes* Cas9	(74)
Cas9	DNA	*Listeria monocytogenes*	*hlyA* gene of *L. monocytogenes*	Electrochemilu-minescence	Higher sensitivity than real-time PCR	0.1 pg/μl, S. pyogenes Cas9, single base resolution	(91)
Cas9	RNA	Zika virus	Viral genome from monkey plasma	Colorimetry	Clinically relevant*	3 fM, 30 min, low cost, Cas9 from NEB, 41°C and 37°C, single-base resolution	(71)
Cas9	DNA	HPVs	L1 fragments of HPVs	Fluorescence	Clinically relevant	1 ng (SiHa gDNA) and 5 ng (HeLa gDNA), 2–3 h, Cas9 from New England Biolabs, 37°C, 72°C, 95°C, 58°C	(72)
Cas9	DNA	*Escherichia coli* O157:H7	*Hemolysin A (hlyA)* gene	Fluorescence	3 orders of magnitude sensitive than RT–PCR kit	4.0 × 10^1^ CFU ml^–1^, 120 min, Cas9 from Novoprotein Scientific, 95°C, 61°C, 72°C, 37°C, 65°C	(73)
Cas9, CAS-EXPAR	DNA, RNA	DNA methylation, *L. monocytogenes* mRNA	*L. monocytogenes hemolysin (hly)* gene	Fluorescence	Less sensitive than isothermal amplifications	0.82 amol, 1 h, Cas9 from New England Biolabs, 25°C, 37°C, 95°C, 55°C, single-base resolution	(126)

Cluc, *Cypridinia* luciferase; dCas9, catalytically dead Cas9; FLASH, Finding Low Abundance Sequences by Hybridization; Gluc, Gaussia luciferase; HPVs, human papilloma viruses; μPAD, microfluidic paper-based analytical device; RT, room temperature; SHERLOCK, specific high-sensitivity enzymatic reporter unlocking; SNP, single nucleotide polymorphism; STOP, SHERLOCK testing in one pot.

*Based on previous information on copy numbers load per ml in urine, patient saliva, and primate and patient serum, we defined the clinical relevance of methods for Zika virus. Zika viral loads have been documented as 202 × 10^6^ copies/ml (365 fM) in urine (127), 3 × 10^6^ copies/ml (4.9 fM) (128) in patient saliva and 2.5 × 10^6^ copies/ml (4.1 fM) (71) and 7.2 × 10^5^ copies/ml (1.2 fM) (129) in primate and patient serum, respectively. If the LOD of methods is less that the viral load in analyte, we considered it as the clinically relevant.

**SARS-CoV-2 CDC assay for RT-qPCR-imputed titers >1000 copy/μl. The methods with LOD in the range of CDC assay are considered as clinically relevant.

***Real-Star Altona, most commonly used molecular test showed sensitivities depending on the Ebola virus: 471 copies/ml (EBOV) and 2871 copies/ml (SUDAN virus).

**Table 2.** CRISPR-Cas based cancer biomarker detection

CRISPR methods	Nucleic acids	Molecule	Type of sample used	Target gene	Detection methods	Detection limit, operation time, cost, point of care (POC), orthologue, temperature (Tm) control, single base resolution	Reference
Cas13	RNA	Transcript tracking- *Gluc*, *Cluc*, *KRAS*, and *PPIB*	*in vitro*	ACTB mRNA	Fluorescence	*Lw*Cas13a, Tm control (37°C)	(22)
SHERLOCK, Cas13	DNA or RNA	Mutations in cell-free tumor DNA	*in vitro*	*BRAF V600E, EGFR L858R*	Fluorescence	Attomolar levels, POC, LwCas13a, Tm control (37°C), single base resolution	(38)
Cas13, PECL-CRISPR	RNA	MicroRNAs	*in vitro*	MiR-17, let‐7	Electrochemiluminescence	1 × 10^−15^ M, low cost, 1 hour and 40 min, POC, LbuCas13a, 37°C and 55°C, single base resolution	(130)
Cas13	RNA	MicroRNAs	*in vitro*	MiR-17, -10b, -155, -21	Fluorescence	4.5 amol, <30 min, POC, LbuCas13a, constant temperature 37°C, single base resolution	(131)
Cas13	RNA	Eight microRNAs	clinical	MiR-19b, miR-20a	Electrochemical	10 nM, 3.5 hour, POC, LwCas13a, RT	(132)
Cas13	DNA	MicroRNAs	clinical	MiR-19b and miR-20a from serum samples	Electrochemical	10 pM, 4 h, low cost, POC, LwCas13a, RT and 37°C, single base resolution	(77)
Cas12a, Cas13	RNA, DNA	MicroRNAs	pig body fluids/ 16s rDNA and 16srRNA pathogenic bacteria	MiRNA-17	Colorimetry	200 copies, 1 nM, 1 h, low cost with detection of *16S rRNA*, AsCpf1, LbuCas13a, Tm control (37°C), single base resolution	(120)
Cas12b	DNA	*BRCA1* and *TP53* SNPs	*in vitro*	*BRCA1* 3232A > G and 3537A > G, *TP53* 856G > A	Fluorescence	Sub-attomolar, AaCas12b, Tm control (37°C), single base resolution	(87)
Cas12a	DNA	Telomeric repeat DNA	clinical	Telomeric repeat DNA	Colorimetry, lateral flow	93.75%, 15 min, POC, LbCas12a, Tm control (37°C)	(86)
Cas12a, E-CRISPR	DNA	TGF-ß1 protein	clinical	TGF-β1 aptamer	Electrochemical	50 Pm, 30 min, low cost electrochemical biosensing, POC, LbCas12a and AsCas12a, Tm control (37°C)	(122)
Cas12a	DNA	Circulating *EGFR* mutations	clinical	*EGFR* mutations (L858R and T790M) from plasma	Fluorescence	0.005%, <3 h, LbCas12a, preamplification and 37°C	(78)
Cas12a	DNA	CfDNA	*in vitro*	*BRCA-1*	Fluorescence, colorimetry	0.34 fM, <30 min, low cost, POC, RT, single base resolution	(82)
Cas9	DNA	Mutations for Duchenne muscular dystrophy	*in vitro* and clinical	DNA samples collected from cells and patients	Electrochemical	1.7 fM, 15 min, dCas9 (University of California, Berkeley MacroLab), Tm control (37°C)	(89)
Cas9	DNA	*EGFR L858R* mutant	*in vitro*	*EGFR*	Electrochemiluminescence	0.1 pg/μl, S. pyogenes Cas9, single base resolution	(91)
Cas9	DNA	CtDNA	clinical	*EGFR*	Electrochemical	0.13 pM, 90 min, Cas9 from Sigma, 90°C, 25°C, single base resolution	(90)
Cas9, Cas9nAR	DNA	*KRAS*	*in vitro*	*G13D* mutation in exon 2	Fluorescence	Zeptomolar (2 copies in 20 μl reaction), 1 h, POC, Cas9 synthesized by GENEWIZ, constant temperature of 37°C, single base resolution	(133)

cfDNA, cell-free DNA; ctDNA, circulating tumor DNA; RT, room temperature; SHERLOCK, specific high-sensitivity enzymatic reporter unlocking; SNP, single nucleotide polymorphism.

Nguyen et al., (2020) extended 3′-end and 5′-end of the crRNA. This led to increased trans-cleavage activity and specificity of LbCas12a, particularly when 3′-end was extended with RNA or DNA. This technique is named the ENHANCE system and can be used to detect DNA: RNA heteroduplex and methylated DNA with high sensitivity. Further, several types of nucleic acids such as ssDNA, dsDNA, and RNA from various samples including HIV, HCV, and SARS-CoV2 could also be detected using ENHANCE technology without the need for further optimization. Specifically, enhanced SARS-CoV2 detection with improved sensitivity and specificity in a fluorescence-based assay and a paper-based lateral flow assay was demonstrated using ENHANCE. However, this technology has limitations as no patient samples were directly used for testing nucleic acid detection so far (19).

To address limitations such as off-target cleavage associated with CRISPR-Cas12a (Cpf1) complex, Kim *et al.* developed a chimeric RNA-DNA guide to reduce off-target cleavage by increasing instability in target DNA binding. The reduction in off-target cleavage is due to reduction in the hybridization energy and increased sensitivity to mismatches. Here, the combination of SpCas9 nickase and chimeric (cr) RNA guided Cas12a led to the development of highly target specific genome editing technique with possible reduction in unwanted off-target cleavages. However, future structural and biochemical studies are warranted on chimeric DNA–RNA recognition of the CRISPR-Cas12a. One possible venue is the improvement of the target DNA cleavage efficiency by modifying amino acid residues in Cas12a, which would increase the safety of *in vivo* target-specific genome editing (20).

### Cas13

RNA-guided ribonucleases (RNases) Cas13 possess four divergent family members (Cas13a–d) and produces numerous cleavage sites in single-stranded areas of target RNA with a specific sequence. Unlike other class 2 effectors, such as Cas9 and Cas12a/Cas12b, Cas13 lacks a DNase domain while it contains two conserved HEPN (complex eukaryotes and prokaryotes nucleotide-binding) domains that cleave RNA (Figure 1A) (21). Cas13 catalyzes both crRNA maturation and RNA-guided single-stranded RNA degradation using two separated catalytic sites in an interdependent manner (22–26). Cas13 cleaves after certain nucleotides and engages in ‘collateral’ cleavage of nearby RNAs after recognition of target RNA (Figure 1B). Thus, Cas13 can be reprogrammed with crRNAs to sense specific RNAs. This crRNA-programmed collateral cleavage could aid to detect the presence of specific RNA *in vitro* by nonspecific degradation of labeled RNA (8) and *in vivo* by inhibition of cell growth and triggering programmed cell death (7). The binding of target RNA at the recognition lobe of Cas13 impacts the dynamics of the spatially distant HEPN1-2 catalytic core, resulting in altered dynamics with respect to the crRNA-bound form, and in the opening of the catalytic cleft. Critical residues in Cas13 (R377, N378 and R973) are involved in selection of target RNA and they rearrange their interactions upon target RNA binding. It was revealed that the alanine mutation of these residues could improve the selectivity of Cas13 as Cas13 was highly specific in discrimination of single-nucleotide polymorphisms (SNPs) in SARS-CoV-2 variants (27,28).

Cas13 enzymes, such as Cas13d and Cas13e are the CRISPR systems available for adeno-associated virus delivery. Compared to Cas13d, Cas13e is highly compact and possesses high efficacy and specificity (29).

Cas13X (also known as Cas13bt3) and Cas13Y were recently identified as subtypes of Cas13. With 775 and 790 amino acids in length, respectively, they are smaller than other Cas13 subtypes, which have 1000–1200 amino acids (29). Surprisingly, species specific alterations in the amino acid residues were noted between Cas13bt3 and PbuCas13b. Cas13bt3 comprises 775 residues which makes it 352-residues shorter than PbuCas13b (1127-residues) and they share very little sequence identity. However, the overall structures of Cas13bt3 and PbuCas13b are similar (30,31). Recent studies on RNA knockdown efficacy of various Cas13 subtypes, such as Cas13X, Cas13Y, Cas13a, Cas13b and Cas13d revealed that Cas13X/Y and Cas13d exhibited significantly higher RNA knockdown efficacy compared to the other subtypes. Specifically, Cas13X/Y and Cas13d showed significant SARS-CoV-2 RNA elimination compared to other subtypes (29,32,33). While Cas13a can collaterally cleave non-target RNAs, Cas13X/Y have relatively weak collateral activity, which requires further investigation (34).

### Cas14

Cas14 proteins with around 400 to 700 amino acids size, are the smallest Cas protein of the Class 2 family. Similar to Cas12, Cas14 has *cis* dsDNA and *cis/*trans ssDNA cleavage activities. Despite its small size, it can target and cleave ssDNA without restrictive PAM sequence requirement. However, it needs T-rich PAM sequences, such as TTTG for dsDNA targeting (35,36). Wei et al., (2021) demonstrated that the strongest trans cleavage activity of Cas14a1 could be triggered by short target RNA (20 nt) and short target ssDNA (20 nt) targets as the activators (37).

## The applications of CRISPR-Cas for sensing

CRISPR–Cas protein driven by guide-RNA is a powerful tool for sequence-specific targeting and detection of nucleic acids including viral RNA and cancer biomarkers. While Cas9 has been most intensely studied in terms of structure and function, Cas13-based detection of nucleic acids has been more exploited in sensing applications. Therefore, in each of the following sections we first discuss the Cas13-based detection methods, followed by those based on Cas12 and Cas9.

### Detection methods for viral RNA/DNA

#### Cas13-based detection of viral RNA and dsDNA

Almost all Cas13-based methods for detection of viral RNA are based on amplification of target RNA and Cas13 collateral cleavage of non-specific RNA (reporter RNA), once Cas13 is bound with the target RNA. The readout from these methods can be in the form of fluorescence, colorimetry, and lateral flow immunochromatography from cleavage of reporter RNA.

**Figure 2.**
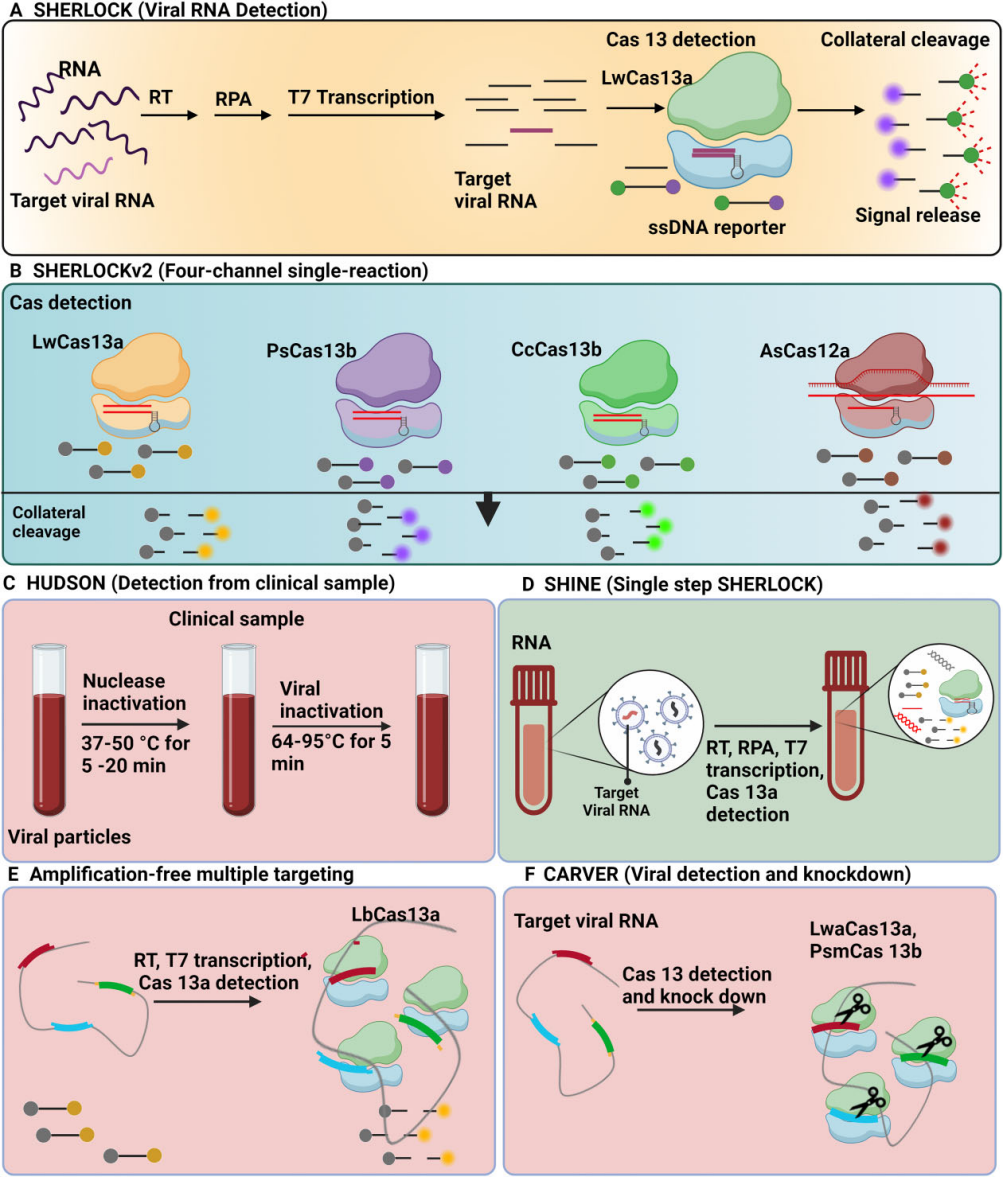
Cas13-based detection methods for viral RNA. (**A**) SHERLOCK is based on the conversion of RNA to DNA by reverse transcription (RT), recombinase polymerase amplification (RPA)-based amplification, and transcription back to RNA for detection by Cas13, and further Cas13-mediated collateral cleavage of a reporter RNA (38). (**B**) SHERLOCKv2 in-sample four-channel multiplexing with orthogonal Cas13 and Cas12a enzymes (39). (**C**) Viral detection directly from bodily fluids using HUDSON (40). (**D**) RPA-based amplification and Cas13 in a single step using SHINE (42). (**E**) Amplification-free multiple crRNAs targeting of Cas13 (46). (**F**) Cas13 for viral detection and knockdown (43).

Gootenberg *et al.* described real-time *in vitro* nucleic acid detection of the target using Specific High-Sensitivity Enzymatic Reporter UnLOCKing (SHERLOCK), based on nucleic acid amplification and dsDNA and RNA detection followed by Cas13-mediated collateral cleavage of a reporter RNA (quenched fluorescent RNA). SHERLOCK is based on the conversion of RNA to DNA by reverse transcription, DNA-based amplification through recombinase polymerase amplification (RPA), transcription back to RNA for detection by Cas13 and fluorescence readout (Figure 2A). SHERLOCK is also used to detect dsDNA by RPA. This method has been used to detect specific strains of Zika and Dengue virus, distinguish pathogenic bacteria, genotype human DNA, and identify mutations in cell-free tumor DNA (cfDNA) (38).

In 2018, four advances integrated into SHERLOCK version 2 (SHERLOCKv2) have been reported: (i) four-channel single-reaction multiplexing with orthogonal CRISPR enzymes allows cost-effective multiple targets detection (Figure 2B); (ii) quantitative analysis of input as low as 2 attomolar; (iii) 3.5-fold increase in signal sensitivity by combining Cas13 with Csm6 that is an auxiliary CRISPR-associated enzyme; and (iv) lateral-flow readout. SHERLOCKv2 could detect Dengue or Zika viruses single-stranded RNA as well as mutations in patient liquid biopsy samples via lateral flow. In this method, the cleavage sequence preference of LwaCas13a, Cas13b from *Capnocytophaga canimorsus* Cc5 (CcaCas13b), LbaCas13a and PsmCas13b was assessed by evaluating their collateral activity across dinucleotide motifs. Then, by leveraging orthogonal dinucleotide motifs, the detection was extended to four targets in FAM, TEX, Cy5 and HEX channels for LwaCas13a, PsmCas13b, CcaCas13b and AsCas12a, respectively (Figure 2B). The advances on in-sample multiplexing via orthogonal based preferences made it possible to perform detection of multiple targets at large scale and more economically (39).

HUDSON (heating unextracted diagnostic samples to obliterate nucleases) is a protocol along with SHERLOCK used to lyse viral particles and to inactivate ribonuclease present in the bodily fluids of patient samples. This step could be accomplished through the application of heat and chemical reduction process (Figure 2C), to detect Zika and Dengue viruses from patient samples in less than 2 hours even at concentrations as low as 1 copy per microliter with a colorimetric readout using lateral flow strips. Furthermore, the method could distinguish the four Dengue virus serotypes, as well as region-specific strains of Zika virus from the 2015–2016 pandemic. This approach eliminates the need for nucleic acid extraction but instead it requires a 30 min incubation time (40).

Qin *et al.* developed an automated microfluidic system and a sensitive fluorometer, coupled with a fully solution-based Cas13 assay (collateral cleavage of fluorescence probes) for detection of Ebola viral RNA. The detection can be done within 5 min, without the need of solid phase extraction. Furthermore, the method needs a small volume of blood sample, that makes the system suitable for finger-prick tests (41).

The outbreak of severe acute respiratory syndrome coronavirus 2 (SARS-CoV-2) triggered the development of a SHERLOCK-based method for detecting SARS-CoV-2 from unextracted samples, named Streamlined Highlighting of Infections to Navigate Epidemics (SHINE). SHINE is an improved HUDSON protocol for detection of SARS-CoV-2 RNA with both paper-based colorimetric and in-tube fluorescent readout. The recombinase polymerase amplification of target and Cas13-based detection is performed in a single step, to simplify the assay, minimizing the risk of cross contamination, and reduce the running time (Figure 2D) (42). It is notable that SHINE’s performance is equivalent to the SARS-CoV-2 CDC assay for reverse transcription quantitative PCR (qPCR) -imputed titers > 1000 copies/μl.

Freije *et al.* developed Cas13-based diagnostic readout using SHERLOCK, that was called as Cas13-assisted restriction of viral expression and readout (CARVER) (Figure 2F). This study investigated whether Cas13 targeting could result in mutations at the site of crRNA targeting. For this, computational analysis was used to identify Cas13 target sites by screening > 350 viral genomes of known species that infect humans. Genome-wide screening of LCMV was used for crRNA design. Later, the results were confirmed experimentally to inhibit the viral replication in three types of ssRNA viruses namely lymphocytic choriomeningitis virus (LCMV), influenza A virus (IAV) and vesicular stomatitis virus (VSV). CARVER allows the CRISPR-Cas effector Cas13 to target numerous mammalian viruses and analyze the effects of targeting and viral response (43). Similarly, Ackerman *et al.* developed Combinatorial Arrayed Reactions for Multiplexed Evaluation of Nucleic acids (CARMEN), a scalable platform that is capable of testing >4500 crRNA–target pairs on a single array. This platform CARMEN-Cas13 allows for validation and comprehensive subtyping of influenza A strains and multiplexed identification of dozens of HIV drug-resistance mutations. Additionally, CARMEN can act at high speed to pin down infected samples for further sequencing to trace the proceeding evolution of the virus thus aids in improved CRISPR-based diagnostics (44). Another study from Welch *et al.* demonstrated the use of first generation of CARMEN called CARMEN v.1, capable of detecting 169 human-associated viruses in 8 samples simultaneously. Specifically, this platform was designed for use during the COVID-19 pandemic, but faced challenges such as obtaining FDA approval, and in the end had a limited application in clinical practice due to the following drawbacks, (i) custom-made imaging chips and readout hardware, (ii) manual intensive 8–10 h workflow, (iii) low-through put sample evaluation. Nevertheless, the method benefits from a smooth workflow and improved sensitivity, without affecting specificity. Once the steps are taken to integrate high-throughput, multiplexed pathogen testing with variant tracking, CARMEN technology can be applied clinically to test respiratory pathogens and their variants (45).

To enhance the sensitivity of Cas13-based diagnosis of RNA, pre-amplification of target RNA is generally required. However, Fozouni *et al.* developed a rapid and amplification-free Cas13 assay for direct detection and quantification of SARS-CoV-2 from nasal swab RNA. This simple and portable method can measure the fluorescence from collateral cleavage of reporter RNA with a mobile phone camera with a compact device that includes low-cost illumination and collection optics. This makes for an appealing tool for point-of-care diagnosis particularly in low income regions. Instead of pre-amplification of target RNA, this method uses multiple crRNAs targeting different parts of the viral genome, that increases the activation of Cas13. It also analyzes the change of fluorescence in real time, instead of a fluorescence measurement only at the endpoint (Figure 2E) (46). In line with this, Shinoda *et al.* developed an amplification-free platform ‘SATORI’ by combining Cas13-mediated RNA detection with microchamber array technology to exhibit several advantages compared to other ssRNA detection methods; (i) SATORI is not affected by amplification errors, (ii) it requires only <5 min for detection, (iii) it is more robust against contaminants such as saliva, therefore can be directly applied to clinical samples without any pre-processing steps, (iv) it is well tolerant to single mismatches between the guide and target sequences. Thus, SATORI serves as a powerful class of accurate and rapid diagnostics for ssRNA viruses such as SARS-CoV-2, Zika, Ebola, HIV and Influenza. When SATORI combined with Cas12a would result in amplification free double-stranded DNA detection that can be applied in diagnosis of DNA virus infections and circulating tumor DNA (47). It has been established that fundamental sensitivity limits of CRISPR-Cas12/CRISPR-Cas13 schemes and their relevant amplification-free assays are determined by kinetic rates. However, the link between them was not well understood until Huyke *et al.* quantified kinetic parameters for various Cas12 and Cas13 enzymes including LbCas12a, AsCas12a, AapCas12b, LwaCas13 and LbuCas13 and their corresponding limit of detection (LOD). The activation of Cas12 and Cas13 enzymes exhibited trans-cleavage catalytic efficiencies between order 10^5^ and 10^6^ M^−1^ s^−1^. This study, aiming at analyzing sensitivity, concluded that cleavage of at least 0.1% of the fluorescent reporter molecules by an activated CRISPR-Cas12/ CRISPR-Cas13 is required for successful detection of the target to further differentiate signal from the background (48). Similar research can be used to examine the sensitivity of various CRISPR assay detection methods, namely those that use tandem nucleases and those that use electrochemical or chemiluminescence-based measures for target identification (49–53).

Loop-mediated isothermal amplification (LAMP) performs highly sensitive nucleic acid amplification in under 20 min with attomolar limits of detection, but often results in non-specific amplification. To overcome this limitation, Chandrasekaran *et al.* developed DISCoVER (for diagnostics with coronavirus enzymatic reporting), which is an RNA extraction free test that linked two different amplification mechanisms for sensitivity with a Cas13-mediated probe for specificity which greatly enhance frequent and on-site molecular diagnostics if mindful steps are undertaken to develop and deploy this assembly (54).

Ren *et al.* developed rapid, specific and sensitive detection of Mycobacterium tuberculosis (MTB) complex PCR-CRISPR-Cas13 detection method (CRISPR-MTB) that could replace old gold standard tuberculosis diagnostic test (55). Similarly, Macgregor *et al.* developed SHERLOCK to detect *Schistosoma japonicum* and *S. mansoni* by a combination of RPA with CRISPR-Cas13 detection through colorimetry or fluorescent readouts. This assay achieved 93–100% concordance with gold standard qPCR detection across all the samples of *S. japonicum*, and achieved 100% sensitivity when compared to qPCR detection of faecal and serum samples of *S. mansoni* (56).

In summary, cleavage of target and non-target RNAs by Cas13 is useful for microbial detection as well as treatment. The methods explained in this section are adapted for detection of microbial RNA and DNA directly from bodily fluids, in multiple-channel single-reaction for multiple detections, and in single step or even amplification-free assay for rapid, easy, and contamination-free detection.

#### Cas12-based detection of viral DNA/RNA

**Figure 3.**
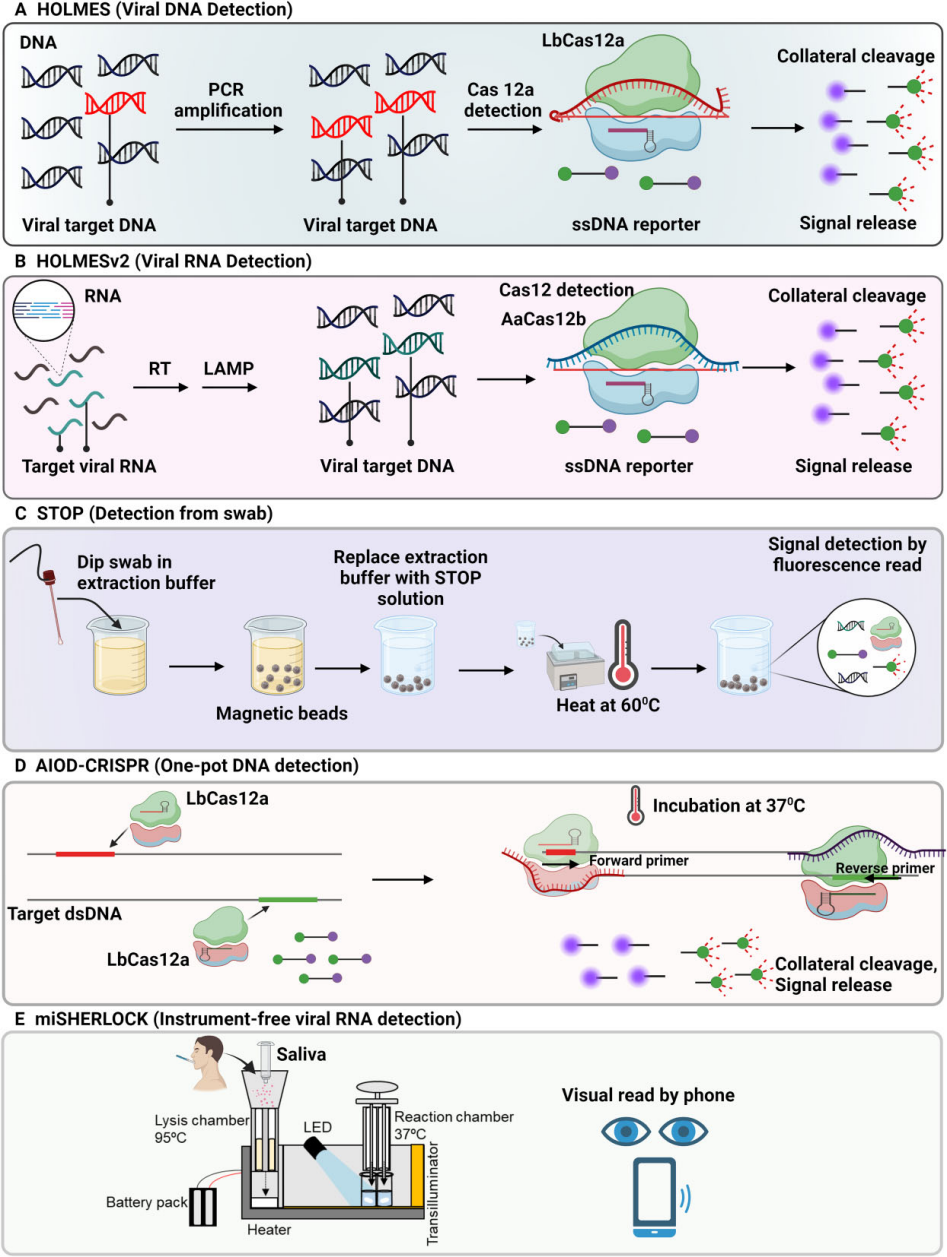
Cas12-based detection methods for viral DNA. (**A**) Detection of viral target DNA using HOLMES (59). (**B**) Detection of viral target RNA using HOLMESv2 (17). (**C**) Simplified viral RNA extraction, isothermal amplification, and Cas12b detection using STOP at a single temperature (60). (**D**) Non-targeted strand displacement at binding sites of Cas12a–crRNA exposes target strand to amplification using AIOD-CRISPR assay (62). (**E**) Instrument-free RNA extraction and concentration from saliva, and one-pot SHERLOCK reaction detects SARS-CoV-2 and variants in reaction chamber, that gives visual fluorescent output (63).

As it was indicated, SHERLOCK is highly sensitive, specific, and suitable method for detection of target RNA. However, for DNA sensing, the shortcoming with SHERLOCK is that *in vitro* transcription of DNA to RNA for sensing DNA sequences is needed, and that brings an extra step to the process. However, Cas12 can directly detect DNA and thereby the *in vitro* transcription of DNA to RNA is not needed for sensing by Cas12. Furthermore, Cas12 possess collateral cleavage activity against non-specific ssDNAs upon the formation of Cas12a–crRNA complexed with target DNA (57,59). Thus, based on these features of Cas12, Cas12-based detection methods, such as one-Hour Low-cost Multipurpose Highly Efficient System (HOLMES) was developed for sensing target viral DNA/RNA, where the quenched fluorescent non-specific ssDNA reporter is used as the probe for readout (Figure 3A) (59).

In 2019, HOLMESv2 was developed for detection of (i) single nucleotide polymorphism (SNP), (ii) virus RNA, human cell mRNA, and circular RNA (Figure 3B) and (iii) quantification of target DNA methylation degree using combined Cas12b detection and bisulfite treatment. In this method, target nucleic acid quantification (fluorescence signal from cleavage of ssDNA reporter) is combined with loop-mediated isothermal amplification, performed in one single step and at a constant temperature of 55°C (15), thus preventing cross-contamination.

SHERLOCK was also adapted for simplified detection of SARS-CoV-2 termed as SHERLOCK testing in one pot (STOP). STOP is a streamlined assay with simplified viral RNA extraction (magnetic bead purification method), isothermal amplification of target, and Cas12b detection. The method is compatible with fluorescence and lateral-flow readouts. Similar to HOLMESv2, it is performed at a single temperature (60°C) in less than an hour, with minimal equipment and without the need of thermocycler (Figure 3C) (60) that makes it cost-effective.

As stated before, Cas12 is able to sense the DNA, and for viral RNA sensing by Cas12, we can skip the conversion of amplified DNA into RNA after the conversion of target RNA to DNA by reverse transcription and DNA amplification. Thus, amplified target DNA will be directly used for detection by Cas12. That is the principle used in a method called as DNA Endonuclease-Targeted CRISPR Trans Reporter (DETECTR) (16). DETECTR has been adapted for SARS-CoV-2 detection from RNA extracted from nasopharyngeal or oropharyngeal swabs. This method includes simultaneous reverse transcription and isothermal amplification of RNA extracted from nasopharyngeal or oropharyngeal swabs in a universal transport medium. It is followed by detection of predefined coronavirus sequences by Cas12 and cleavage of reporter molecules for fluorescence/lateral flow readouts as the proof of virus detection (61). This method is deemed to be superior to the reverse transcription qPCR assay for SARS-CoV-2 by the US Centers for Disease Control and Prevention, as in comparison, it provides a faster and visual readout (61).

Furthermore, Ding et. al., (2020) developed an all-in-one dual CRISPR-Cas12a (AIOD-CRISPR) assay that is one-pot (mixed components for amplification and CRISPR detection) detection method for SARS-CoV-2 that is performed at a single temperature of 37 °C (Figure 3D). In this case, dual crRNAs specific to the SARS-CoV-2 nucleoprotein gene without PAM sequence limitation were introduced for dual CRISPR-based nucleic acid detection. For that, a pair of Cas12a–crRNA generated using two individual crRNAs, binds to two different sites in the target sequence and displaces non-targeted strands. The strand displacement exposes the binding sites of the Cas12a–crRNA complexes to the forward and reverse primers present in one pot reaction mixture. The mixture incubation at ∼37 °C initiates the recombinase polymerase amplification. Furthermore, upon Cas12a–crRNA binding with the amplified target sites, the Cas12a endonuclease creates strong fluorescence signal by cleaving the ssDNA reporters (62). In this method, the amplified products continuously trigger the Cas12a collateral cleavage activity and thereby strengthening the fluorescence signal.

Given the constant evolution of new SARS-CoV-2 variants, it is critical to quickly adapt the diagnostics. Minimally instrumented SHERLOCK (miSHERLOCK) is a one-pot SHERLOCK reaction with RNA paper-capture method compatible with *in situ* nucleic acid amplification and Cas12a detection with visual fluorescence readout. The miSHERLOCK includes lysis chamber and reaction chamber. In lysis chamber, the patient introduces saliva to the sample preparation column followed by addition of lysis buffer and heating to 95°C (3–6 min) for the elimination of viral particles and false-positive signal related to the nucleases present in saliva. Saliva flows through a polyethersulfone membrane which concentrates and accumulates viral RNA. Then, the flow column needs to be transferred to the reaction chamber to release the polyethersulfone membrane as well as stored water into freeze-dried pellet of one-pot reaction. The result can be visualized after 55 min directly or by a smartphone that quantifies fluorescent signal. The method is capable of concurrent universal detection of SARS-CoV-2 as well as specific detection of B.1.1.7, B.1.351 or P.1 variants by designing guide RNAs and RPA primers for universal detection as well as specific detection of variants. It is instrument-free, and it has built-in sample preparation from saliva, room temperature stable reagents, battery-powered incubation, simple visual and mobile phone output interpretation with a LOD of 1000 copies/ml that matches US Centers for Disease Control and Prevention reverse transcription qPCR assay for SARS-CoV-2 (Figure 3E) (63).

**Figure 4.**
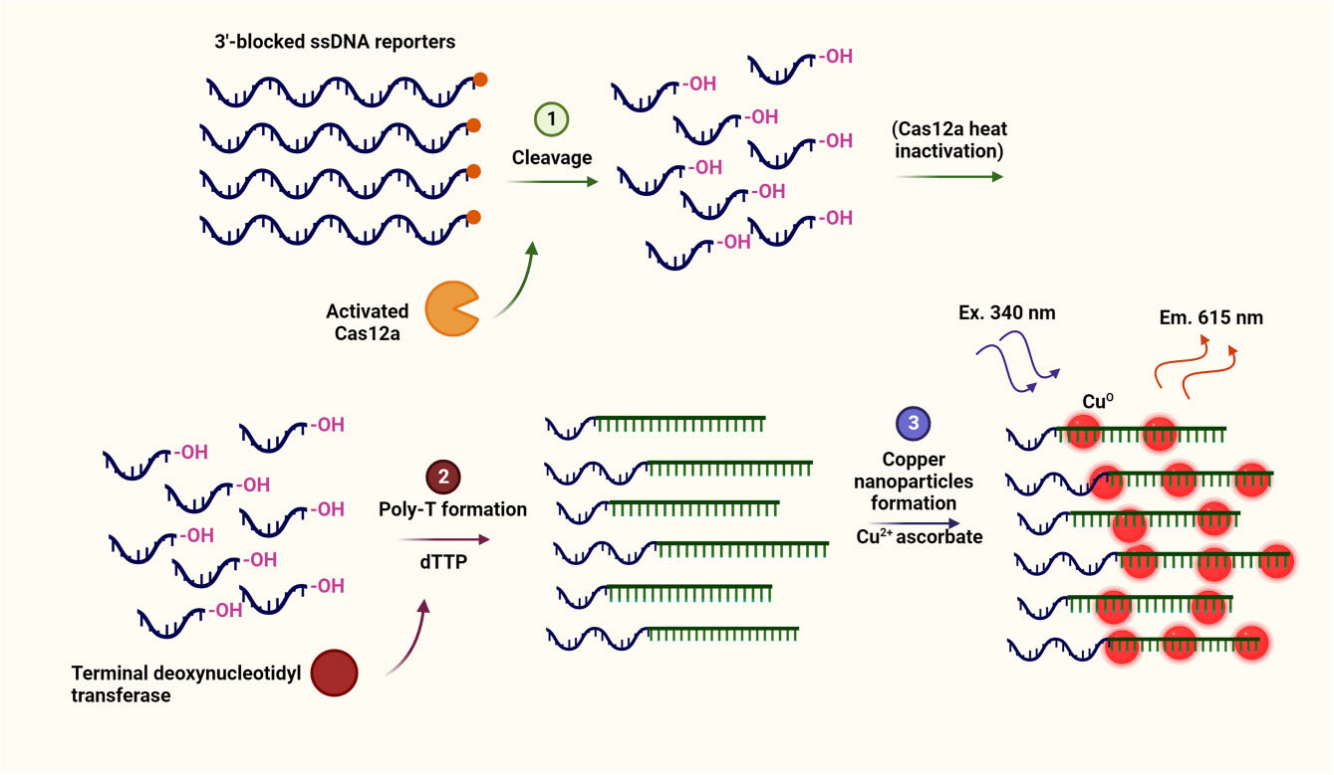
Cas12a activated nuclease poly-T reporter illuminating particles (CANTRIP) for DNA detection. After target DNA recognition by Cas12a, ssDNA reporter oligos with blocked 3′-ends are cut into smaller ssDNA fragments, generating neo3′-hydroxyl moieties. Terminal deoxynucleotidyl transferase incorporates dTTP nucleotides into these fragments and produces poly(thymine)-tails that function as the scaffolds for the formation of copper nanoparticles with a bright fluorescent signal (64).

A DNA detection assay termed Cas12a Activated Nuclease poly-T Reporter Illuminating Particles (CANTRIP) was developed using combined function of two enzymes, terminal deoxynucleotidyl transferase and Cas12a for sensing anthrax lethal factor gene (Figure 4). The method generates copper nanoparticles that are visible by the naked eyes under UV-light. After target DNA recognition by Cas12a, ssDNA reporter oligos with blocked 3′-ends are cut into smaller ssDNA fragments, generating neo3′-hydroxyl moieties. Deoxynucleotidyl transferase incorporates dTTP nucleotides into these fragments and produces poly(thymine)-tails that function as the scaffolds for the formation of copper nanoparticles with a bright fluorescent signal. The limitation with this method is that, unlike SHERLOCK, HOLMES and DETECTR, target amplification in this method is not possible, since the amplification primers would be used by deoxynucleotidyl transferase for poly-T formation (64).

**Figure 5.**
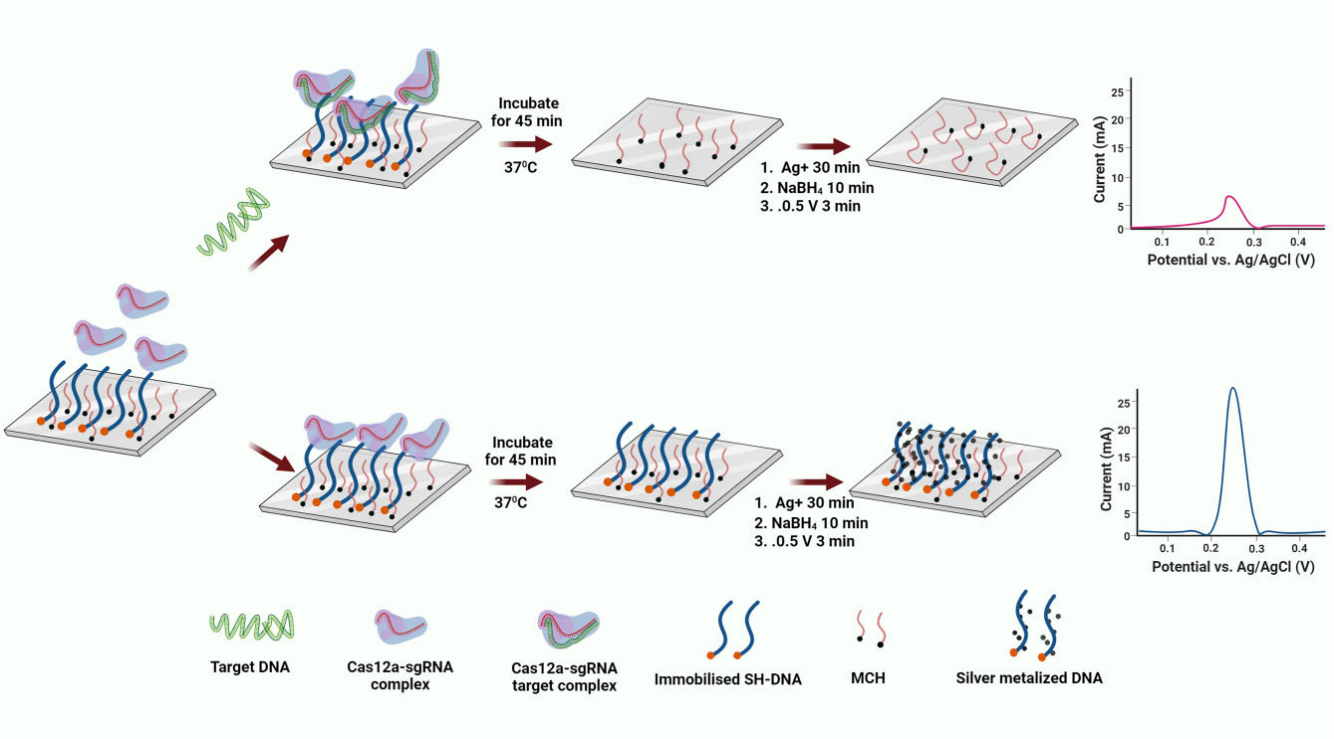
Electrochemical luminescence biosensor to detect antimicrobial resistance genes in bacteria. ssDNA immobilized on the electrode is cleaved by Cas12a in the presence of the target gene. Subsequent addition of Ag^+^ and NaBH_4_ seeds the silver metallization, followed by double metallization when a potential is applied, that yields a minimized electrochemical signal. Once the target gene is absent, ssDNA on the electrode is not cleaved, and yields a higher electrochemical signal (51).

When it comes to readout, besides fluorescence-based detection methods such as SHERLOCK, other approaches such as electrochemical-based detection of nucleic acids were also established using Au nanoparticles (AuNPs). Lee *et al.* conjugated AuNPs with methylene blue (MB) (electrochemical probe) and immobilized the conjugate on working electrode via SH-ssDNA-biotin. The ssDNA was non-specifically cleaved by Cas12a in the presence of Dengue fever viral RNA leading decreased electrochemical signal from MB-AuNPs (65). The approach exhibited an ultrasensitive detection of viral RNA (as low as 100 fM) without any amplification as the PCR based detection method of dengue virus could be able to detect only 100 copies/ml of plasma (66). A novel electrochemical sensor platform was developed for the rapid and sensitive detection of nucleic acids. The results revealed that lower surface coverage density and incompact morphological structure are the reason for the lower steric hindrance effect and higher efficiency Cas12a cleavage of the hairpin DNA reporter for improved analytical sensitivity. Apart from that this platform can also detect target complex matrices providing a potential tool for POC diagnostics (67). In another study, an electrochemical luminescence biosensor was developed with incorporation of silver metallization to electrochemical CRISPR-based biosensor to detect pathogenic genomes, such as antimicrobial resistance gene in bacteria. In this method, ssDNA immobilized on the electrode is cleaved by Cas12a in the presence of target gene. Subsequent addition of Ag^+^ and NaBH_4_ seeds the silver metallization followed by double metallization when a potential is applied, that yields a minimized electrochemical signal. However, once the target gene is absent, ssDNA on the electrode is not cleaved, and that yields a higher electrochemical signal (51) (Figure 5). These ultrasensitive and amplification free electrochemical-based detection methods produce strong enough signal and eliminate the need for amplification of target DNA/RNA.

**Figure 6.**
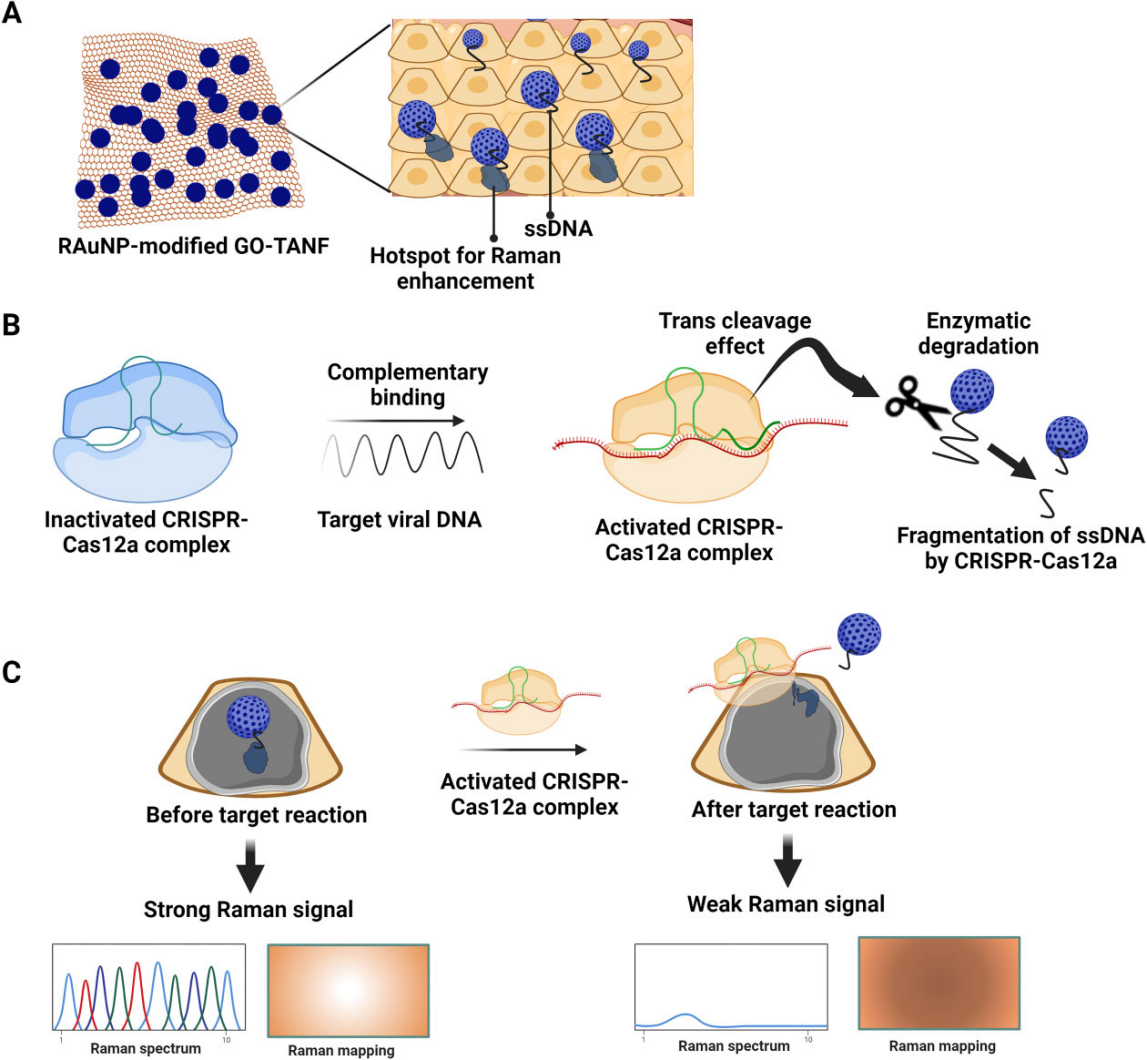
ssDNA-immobilized Raman probe-functionalized AuNPs (RAuNPs) on GO/triangle Au nanoflower array for multiviral DNA detection. (**A**) Graphene oxide (GO)/triangle Au nanoflower (GO-TANF) with RAuNP enhances the surface-enhanced Raman spectroscopy (SERS) signal by generating a hot spot between GO-TANF and RAuNP. (**B**) Trans-cleavage effect of activated CRISPR-Cas12a by target viral DNA. (**C**) RAuNPs on the GO-TANF separates from the surface due to the cleavage of ssDNA, results in reduction of maximized SERS intensity (68).

In order to enhance the sensitivity of amplification-free Cas12a-based detection, a Raman-sensitive system was fabricated using ssDNA-immobilized Raman probe-functionalized AuNPs (RAuNPs) on GO/triangle Au nanoflower array (Figure 6). This method could detect multiviral DNA (hepatitis B virus (HBV), human papilloma viruses (HPVs) HPV-16, HPV-18) with an extremely low detection limit of 1 aM to 100 pM, without amplification (68). Meanwhile the analytical sensitivity of most commonly used PCR-based assays for HBV DNA (Xpert assay HBV DNA platforms, Abbott RealTime HBV test, Roche Cobas AmpliPrep/Cobas TaqMan HBV test, Roche Cobas TaqMan HBV test with the HighPure system, Qiagen Artus HBV and Aptima Quant HBV tests) is 10 IU/ml for the plasma sample (69). In case of HPV, the sensitivity using digene HC2 HPV DNA test from Qiagene is 100 000 copies of hrHPV per ml of sample, or 100 copies of hrHPV per μl of sample (70).

Generally, both Cas13 and Cas 12 detection techniques described here are mostly based on signal release from collateral cleavage activity of Cas12/Cas13 against ssDNA/ssRNA reporter. However, nucleic acid detection using Cas12 is more convenient, since T7 transcription step for converting amplified DNA to RNA before Cas13 detection is omitted in Cas12-based detection assays. Furthermore, recent advances, including one-pot assay at a constant temperature and ultrasensitive amplification-free detection, reduce the risk of contamination, dual crRNAs strengthen the signal without PAM sequence limitation and instrument-free detection makes the technology more accessible.

#### Cas9-based detection of viral and bacterial DNA/RNA

**Figure 7.**
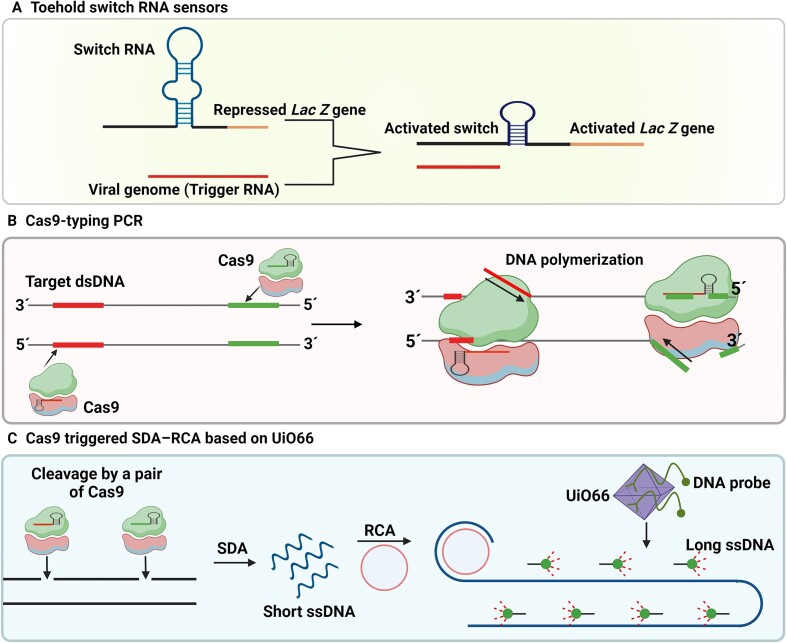
Cas9-based detection methods for viral nucleic acid. (**A**) Combined isothermal RNA amplification with toehold switch RNA sensors controlling translation of *LacZ* (color change) through binding with Zika genome as the trigger RNA (71). (**B**) Cas9-typing PCR. Target DNA cleaved by Cas9-sgRNAs followed by releasing two single strands with free 3′ ends that anneal with a pair of oligonucleotides for polymerization of DNA from the free 3′ ends and further PCR amplification by universal primers (72). (**C**) Cas9 triggered SDA–RCA method based on UiO66 platform. A pair of Cas9: sgRNA complex cleave one strand of target DNA, strand displacement amplification (SDA) extends at the nick while displacing the original DNA strand and producing some short-ssDNAs. Then, a long-ssDNA copy of the circular probe with repeat sequences is synthesized through rolling circle amplification (RCA) once the circular probe hybridized to 3′ of short-ssDNA. Next, fluorescent probes leave UiO66 and hybridize with long-ssDNA, that produces fluorescence signal (73).

In case of Cas9, there is no collateral activity against non-specific ssDNA/ssRNA. This makes Cas9 different from the Cas12 and Cas13 in sensing approaches (71). A simple, field ready sample processing workflow was demonstrated by linking isothermal RNA amplification to toehold switch RNA sensors to detect clinically relevant concentrations of zika virus sequences and with specificity against dengue viral sequences (Figure 7A). When coupled with a novel CRISPR-Cas9-based module, the sensor can distinguish between distinct viral strains with single-base resolution (71).

A novel Cas9-based DNA detection method, named CRISPR-Cas-typing PCR version 4.0 (ctPCR4.0) was developed to detect L1 fragments of HPVs from clinical samples. In this method, the target DNA is cleaved by a pair of Cas9-sgRNAs. It is followed by releasing two single strands with free 3′ ends that anneal with a pair of oligonucleotides for polymerization of DNA from the free 3′ ends, further PCR amplification by universal primers, and finally fragment detection using qPCR or on agarose gel electrophoresis (Figure 7B) (72). The method addresses the limitations of PCR for primer design and nonspecific amplification by introducing CRISPR-Cas system into PCR.

For bacterial detection, the strand displacement amplification (SDA)–rolling circle amplification (RCA) combined with Cas9 was applied for fluorescence detection of *E. coli* O157:H7 using the metal–organic framework (MOF) UiO66 platform (Figure 7C). UiO66 is a cubic framework of cationic Zr_6_O_4_(OH)_4_ nodes and 1,4-benzenedicarboxylate linkers, that adsorbs fluorophore-labeled ssDNA and quenches the fluorescence. Upon target DNA recognition and cleavage by Cas9, short ssDNAs are produced from SDA, that are used as the primer for RCA, leading to synthesis of a long DNA chain. In the presence of long ssDNA, the fluorescence ssDNA probes that are bound with UiO66, depart from UiO66 and hybridize with the long-ssDNA, leading to the fluorescence recovery (73).

**Figure 8.**
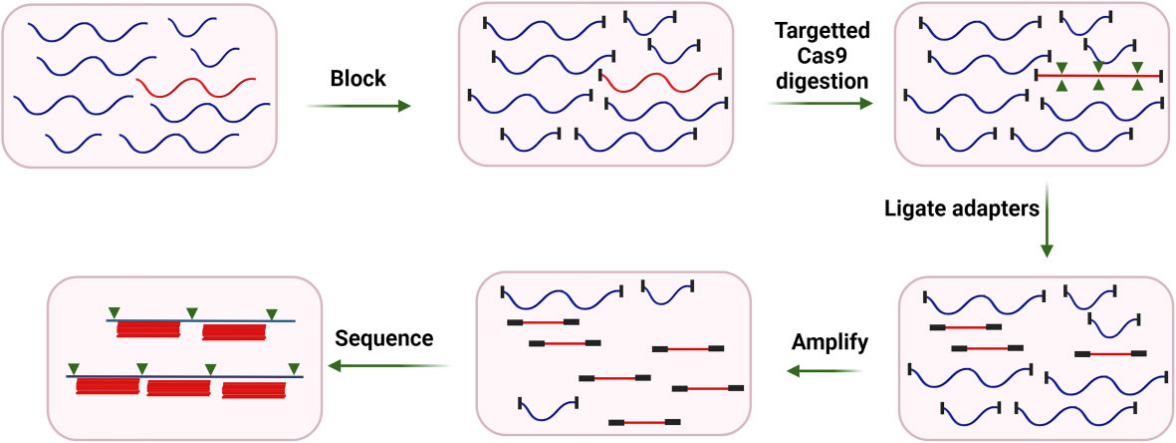
Finding Low Abundance Sequences by Hybridization (FLASH) for detection of antimicrobial resistance genes. Cas9 guide RNAs cleaves the sequences of interest into fragments for Illumina sequencing. Then, the cleaved products are ligated with universal sequencing adapters. The amplification step ensures the enrichment of target sequences for binding to the sequencing flow cell.

Quan *et al.* also introduced a next generation CRISPR-Cas diagnostic method termed Finding Low Abundance Sequences by Hybridization (FLASH) for detection of antimicrobial resistance genes (Figure 8). The FLASH technique with a set of Cas9 guide RNAs cleaves the sequences of interest into fragments for Illumina sequencing. Then, the cleaved products are ligated with universal sequencing adapters. The amplification step ensures the enrichment of target sequences for binding to the sequencing flow cell (74). This method is very precise and allows for a high level of multiplexing (thousands of targets analyzed simultaneously).

Unlike Cas13 and Cas12 detection processes that are based on collateral cleavage activity against non-specific DNA, Cas9 does not have this property and Cas9 detection systems, such as toehold-based sensor, Cas9-typing PCR, and Cas9 triggered SDA-RCA are based on cleavage of target DNA, as described in here.

#### Cas10-based detection of viral RNA

Like Cas13, Cas10-based type III CRISPR system is also used to detect viral RNA. Sequence-specific detection of viral RNA was observed with Cas10, which makes it unique among other detection methods. For a sensitive, specific and rapid diagnostic of SARS-CoV-2, type III CRISPR Csm complex that triggers Cas10-mediated polymerase activity was used. As a result, this study (75) concluded that viral RNA recognition by the type III CRISPR-Cas complex stimulated Cas10-mediated polymerase activity which resulted in the generation of pyrophosphates, protons, and cyclic oligonucleotides within 1–30 min, which can be detected using colorimetric or fluorometric methods (75). Additional modifications, such as re-designing primer sets could increase the efficacy of reverse-transcription LAMP at 55°C, or using a more thermostable RNA polymerase increase the efficacy of T7 transcription-Csm detection at 65°C, result in a successful integrated one-pot diagnostic (75). Although type III-A systems have unique dual DNA and RNA targeting complex mechanisms, several questions remain unanswered, such as how crRNAs translocate from Cas6 endonuclease to the Cas10 complex as Cas6 endonuclease needs to cleave and liberate the crRNA to be transferred to Cas10, and how the Cas10 complex search for the transcripts for the complementary sequence to the crRNA (76).

#### Cas14-based detection of viral and bacterial DNA/RNA

Although only a few studies so far reported on CRISPR-Cas14, this system appears superior to other Cas13 systems. Harrington et al., (2018) first combined the use of CRISPR-Cas14 system with the DETECTR technique by designing gRNA targeting the HECT and RLD Domain Containing E3 Ubiquitin Protein Ligase 2 (*HERC2*) gene in human saliva collected from blue-eyed single nucleotide polymorphisms (SNP) individuals. This result showed that Cas14 was more advantageous than Cas12, where Cas12 failed in recognition of the blue-eyed SNP. It seems to be a cost-effective method for screening mutations of pathogens and a great opportunity for mapping the candidate genes associated with various pathogens (36).

Wei *et al.* developed Aptamer-based Cas14a1 Biosensor (ACasB) to detect a large variety of pathogenic microorganisms by simply changing the guide RNA sequence. The biosensor does not need amplification and nucleic acid extraction. In this case, *S. aureus* specific aptamer was hybrid with a blocker DNA. Upon addition of *S. aureus*, the blocker was released upon bacteria-aptamer binding, and further activation of Cas14a1 protein when bound with sgRNA generates fluorescence intensity. This method is more advanced than qPCR-based detection, which exhibited 100% accuracy and can differentiate live and dead bacteria in complex samples in food safety and disease diagnosis (35).

### Genotyping by single nucleotide polymorphism (SNP)

Over one and half million SNPs that are related to risk of disease are found in the human genome. There are some Cas-based methods available for detection of these SNPs.

Both extracted genomic DNA and saliva from human individuals have been used for PCR amplification and then HOLMES assay could determine both homozygous and heterozygous genotypes. HOLMES could also easily and rapidly detect human SNP (rs1014290) genotypes (59).

HOLMESv2 was used to examine the SNP locus of rs5028 from the human cell line HEK293T. Since there was no PAM sequence near the locus, two distinct approaches of asymmetric PCR and loop-mediated isothermal amplification were used for target amplification. Asymmetric PCR amplifies the sense strand from the original DNA to a greater extent than the anti-sense strand. It is used in hybridization probing when only one of the two strands is required. Asymmetric PCR produces ssDNA, and Cas12b could distinguish the SNP locus without any PAM sequence. However, in case of loop-mediated isothermal amplification, the PAM sequence was necessary for distinguishing SNP locus by Cas12b, so it was introduced in amplification primers (17).

### Detection methods of DNA/RNA biomarkers in cancer

#### Cas13-based detection of DNA/RNA biomarkers in cancer

As stated before, Cas13-based SHERLOCK can detect cell-free tumor DNAs including BRAF V600E and epidermal growth factor receptor (EGFR) L858R. However, its limitation is the additional step of *in vitro* transcription of cell-free tumor DNA to RNA for sensing by Cas13 (38).

**Figure 9.**
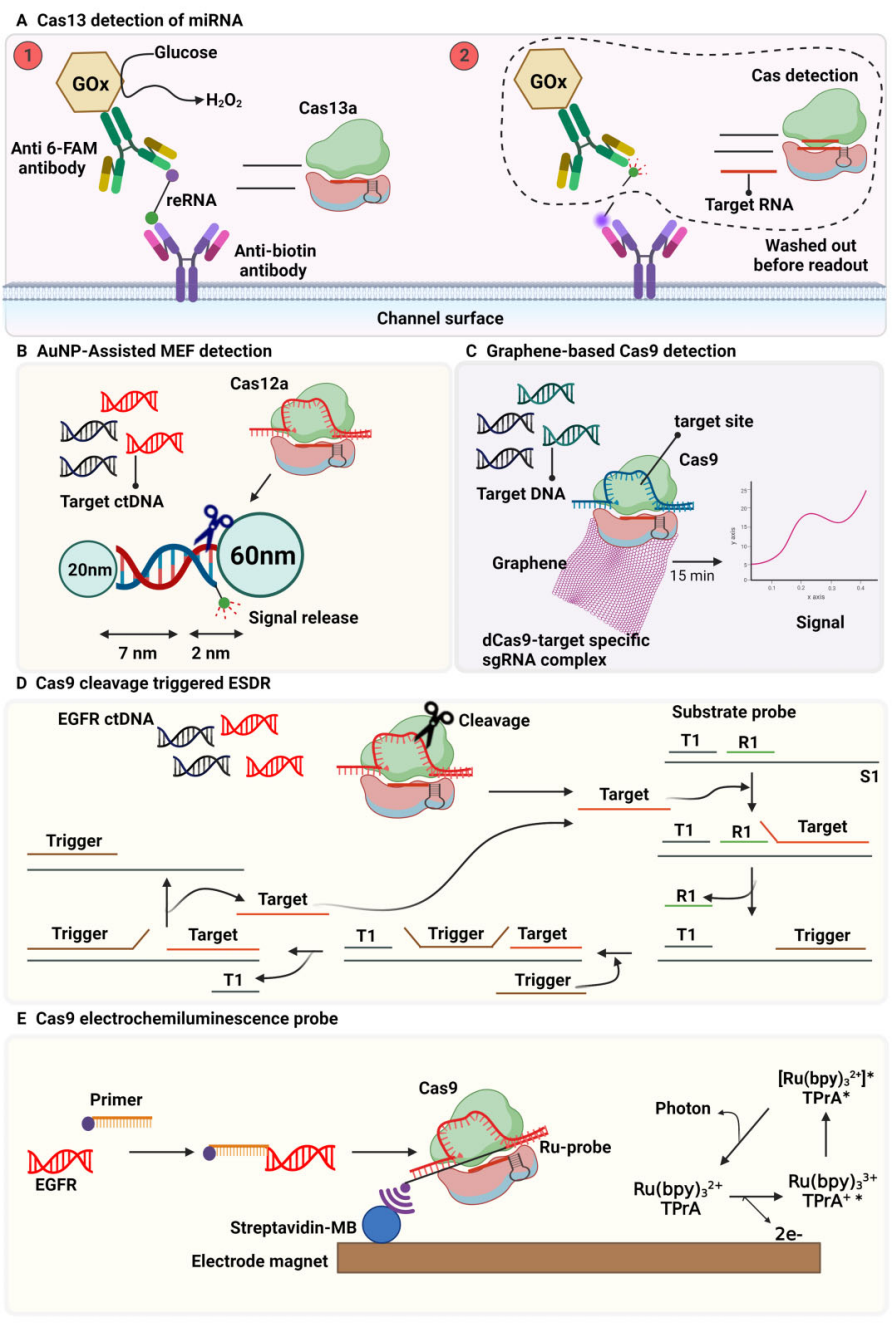
Cas-based detection of cancer biomarkers. (**A**) Cas13-based amplification-free miRNA diagnostics. Upon introducing miRNA and crRNA/Cas13 complex in number 2, the enzyme cleaves the immobilized reporter RNAs (reRNAs), that remobilizes the glucose oxidase (GOx)-conjugated antibodies. The readout can be conducted using glucose solution catalyzed by GOx (77). (**B**) SsDNA is cleaved by Cas12a and induces MEF in the presence of cell-free tumor DNA (cfDNA) and its complex formation with Cas12a (82). (**C**) Cas9 graphene-based detection of mutation related to Duchenne muscular dystrophy. The gene-targeting capacity of Cas9 with the sensitive detection power of a graphene-based field-effect transistor (gFET) is combined (89). (**D**) Cas9 cleavage triggered ESDR for ctDNA detection on a 3D graphene/AuPtPd nanoflower biosensor. Cas9/sgRNA cleaves the target DNA that triggers the ESDR in the triplicate DNA (T1, R1, and S1) and caused release of initial target sequence (90). (**E**) Cas9 electrochemiluminescence probe for DNA detection. SgRNA, dCas9 and labeled Ru-probe assembled into a dCas9-elecrochemiluminescence (ECL) probe. Labeled primer is for PCR amplification to obtain labeled dsDNA products, recognized by the dCas9-ECL probe and captured by streptavidin-modified magnet beads. Then, upon addition of TPrA, the excited-state form of [Ru(bpy)_3_^2+^]^∗^ is produced at the diffusion layer of an electrode, that turns into the ground-state by photon emission and gives ECL signal (91).

MicroRNAs (miRNAs) are composed of 20–22 nts and play an essential role in regulation of gene expression related to biological processes. Certain cancer types are linked to the aberrant regulation of specific miRNAs. Cas13-powered microfluidics integrated electrochemical biosensor was introduced for on-site detection of microRNAs. It can quantify the potential tumor markers, microRNA miR-20a and miR-19b with a detection limit of 10 pM without nucleic acid amplification, which shows a good agreement with the standard reverse transcription-qPCR method. In this method, anti-biotin antibodies, biotin, 6-FAM-labeled reporter RNA (reRNA) and glucose oxidase (GOx)-labeled anti-fluorescein antibody are immobilized on the surface of the biosensor. Upon introducing miRNA and its incubation (37°C) with crRNA/Cas13 complex in a microchannel, Cas13 cleaves immobilized reRNAs. This remobilizes the GOx-conjugated antibodies. After washing all unbound GOx-labeled antibodies, the readout can be conducted using glucose solution. Glucose oxidation, catalyzed by GOx, produces H_2_O_2_ that can be detected in an electrochemical cell (Figure 9A). As it was shown in panel 2 in figure 5A, the target microRNA activates Cas13 to cleave the bound reRNA, leading to removal of the GOx-labeled antibody, thereby reducing the amperometric signal (77). In this method, it is expected that immobilization of Cas/crRNA complex on the surface might be helpful to beneficially reduce the LOD. Overall, detection of cancer biomarkers including cell-free tumor DNAs and microRNAs can be performed using Cas13. These methods rely on fluorescence and electrochemical readouts.

#### Cas12-based detection of DNA biomarkers in cancer

EGFR is a transmembrane receptor tyrosine kinase, that induces the growth and proliferation of cancer cells. Thus, the detection of EGFR mutations as circulating tumor DNA (ctDNA) in body fluids, such as blood plasma, can be applied in clinical decisions related to targeted EGFR therapies. Tsou *et al.* (2020) used the collateral cleavage activity of Cas12a against non-specific ssDNA reporter as a simple test with fluorescent readout to sensitively detect circulating tumor-DNA via EGFR point mutations in plasma (78).

Cell-free DNA, including circulating tumor-DNA originate mainly from apoptosis but also from necrosis and secretion (79). Levels of circulating tumor-DNA correlate with cancer progression (80). The use of blood plasma for DNA analysis is considered to be minimally invasive and suitable for repeated sampling. Such applications include screening, diagnostics, prognostics, monitoring treatment efficacy as well as early detection of minimal residual disease, treatment resistance, and relapse (81). Cas12a fluorescent-based amplification-free biosensor was developed to detect circulating tumor-DNA by AuNP-assisted metal-enhanced fluorescence. The method includes two different sized AuNPs (20 nm and 60 nm) connected by a 7 nm dsDNA and a 2 nm ssDNA (reporter) that is cleaved by Cas12a in the presence of circulating tumor-DNA and induces metal-enhanced fluorescence. Quenching fluorescence occurs in the absence of circulating tumor-DNA (Figure 9B). On the other hand, a color change from red to purple as well as shift in absorbance from 532 (20-AuNP) and 531 nm (60-AuNP) to 540 nm (nanoparticle complex via DNA complementary binding) were observed using colorimetric analysis when a target DNA binding CRISPR-Cas12a complex to the 20- and 60-AuNP binding complex takes place. However, the color change can be reverted when there is initiation of dissociation of AuNP and ssDNA indicating that color and absorbance peak changes dependent on trans-cleavage reaction of the CRISPR-Cas12a by the target DNA and therefore can be used as an alternative detection method (82).

The non-specific cleavage activity of different types of Cas12a (LbCas12a and AsCas12a) against ssDNA anchored on AuNPs has been compared, and it was found to be highly dependent on length and density of ssDNA (83). Another study found that non-specific cleavage of dsDNA probes with staggered ends by Cas12a was more effective than hairpin and ssDNA probes. This scenario could be explained by the orientation of the dsDNA probe, which alters accessibility of Cas12a (84).

Telomerase activity was found to be a good proxy for cancer detection. Telomerase is an enzyme that adds new DNA repeats into telomeres (chromosomal extremities), thus maintaining the stability of chromosomal ends, playing an essential role in stem cell maintenance/renewal. Negligible telomerase activity was found in most normal somatic cells except for germ cells. However, around 85% of primary tumors shows significant telomerase activity (85). Cas12a and AuNP-DNA probes were used for colorimetric detection of telomeric repeat DNAs. In this method, ssDNA is added to the reaction, containing a terminus complementary to DNA probes fixed on AuNPs. When there is no telomeric repeat DNAs in the sample, ssDNA terminus is hybridized with DNA probes on AuNPs, and cross-links AuNPs to form aggregates. However, once Cas12a/crRNA complex recognizes the telomeric repeat DNAs, ssDNA is degraded by Cas12a, thus, AuNPs remain in dispersed form, resulting in red color (86).

Compared to Cas12a-based detection platforms, Cas12b-mediated DNA detection (CDetection) strategy is highly sensitive. The tgRNA-programmed Cas12b-mediated DNA detection (CDetection) could detect the human *BRCA1* gene with a SNP (3232A > G) responsible for breast cancer. This method amplifies the BRCA1 3232A > G and wild-type allele from DNA of human breast cancer cells, using recombinase polymerase amplification. It further differentiates the SNP with strong fluorescent signal from wild-type allele with near-background signal with allelic fractions as low as 1% (87). This CDetection strategy is considered advantageous over Cas12a-DETECTR as it can detect dsDNA directly without an extra step as in Cas-SHERLOCK or Cas14-DETECTR platform. However, this technique has the following limitations: i) Due to inadequate purity and integrity, clinical specimen detection may be difficult, ii) For CRISPR-Cas-based nucleic acid detection technologies, the RPA pre-amplification step is critical for producing a strong signal and avoiding false positives (87).

Table 2 demonstrates that the Cas12 based detection of cancer DNA/RNA biomarkers could be based on fluorescence, electrochemical, and colorimetric readouts. Colorimetric based detection, without need of any sophisticated visualization instruments, is the most promising route to make cancer diagnostics more accessible.

#### Cas9-based detection of DNA biomarkers in cancer

A nanopore-based diagnostic tool for direct detection of DNA was also developed using DNA sensing capability of Cas9. Catalytically deactivated dCas9 variant used in this work is not able to cut DNA, but instead it remains bound at a user-defined binding site, making it an interesting tool for biosensing (88).

Hajian *et al.* used the gene targeting property of dCas9 to detect two distinct mutations at exons commonly deleted in individuals with Duchenne muscular dystrophy. They immobilized Cas9 on a graphene-based field-effect transistor (gFET), to combine gene-targeting of Cas9 and sensitive detection power of gFET. The method provides a rapid, facile, amplification free, and selective detection of target sequence contained within intact genomic DNA (Figure 9C) (89).

The abundance of ctDNAs is low in peripheral blood and the background of wild-type DNA is very high. That makes specific and precise measurement of ctDNA challenging. A novel three-dimensional graphene/AuPtPd nanoflower electrochemical biosensor was developed for detection of EGFR ctDNA based on Cas9 cleavage-triggered entropy-driven strand displacement reaction (ESDR). ESDR employs several ssDNA molecules thermodynamically driven forward by the entropic gain of liberated molecules in an enzyme-free reaction. In this method, the Cas9/sgRNA complex cleaves the target DNA, that triggers ESDR creating triplicate DNA substrate probe (T1, R1 and S1 chains) (Figure 9D). Addition of trigger ssDNA causes release of initial target sequence that is the promoter for the next cycle, resulting in a high variation in signal (90).

The electrogenerated specific chemiluminescence reaction on the surface of a working electrode (WE) is called elecrochemiluminescence (ECL). The transfer of high energy electrons between the illuminating substance and WE forms the excited state illuminator that can turn into the ground state by emission of photons. Catalytically dead Cas9 (dCas9) was used as the universal ECL probe for single base specific and sensitive sensing genome of *Listeria monocytogenes* and EGFR L858R mutation. In this method, the sgRNA, dCas9, and labeled Ru-probe are assembled into a dCas9-ECL probe. A labeled primer is used for PCR amplification to obtain labeled dsDNA products. The products are further recognized by the dCas9-ECL probe and captured by streptavidin-modified magnet beads. Then, upon addition of tripropyl amine (TPrA), the excited-state form of [Ru(bpy)_3_^2+^]^∗^ is produced at the diffusion layer of an electrode, that turns into the ground-state by photon emission and gives ECL signal (Figure 9E) (91).

As shown in Table 2, the detection of cancer biomarkers using Cas9 is mostly based on Cas9 binding with target DNA and cleavage of target DNA on electrochemical sensing platforms.

## Challenges and perspectives

Rapid detection of nucleic acids with high sensitivity and single-base specificity on a portable platform would be ideal in diagnostics and to monitor disease and treatment. With the advanced methods reviewed herein, the steps were already taken towards the development of a powerful tool that reduce personnel time, required equipment, assay time, increase adequate sensitivity (sub-attomolar), and specificity of detection. These steps are crucial for improving the accessibility of diagnosis, and for monitoring the patients in relation to disease progression and treatment efficacy. Now SARS-CoV-2 RNA DETECTR assay and SHERLOCK CRISPR SARS-CoV-2 kit are not FDA cleared or approved, but they are authorized by FDA under an Emergency Use Authorization (EUA) for use by authorized laboratories (92).

In the following section, we discuss the requirements for an optimal detection method, challenges with the currently used methods and possible solutions for these challenges.

### One-pot versus two-steps methods

SHERLOCK is normally performed in two steps: amplification of the target and Cas-based nucleic acid detection (61). However, due to the RNA extraction and multiple liquid-handling steps, this method is complicated and increases the risk of cross-contamination in the samples compared to those methods that are applied in point-of-care testing. In contrast, one pot strategy is used in streamlined SHERLOCK testing or STOP that combines simplified extraction of viral RNA, isothermal amplification, and CRISPR-mediated detection of SARS-CoV-2. The test is fast and can be performed in less than one hour, at a single temperature, and with minimal equipment. Furthermore, the sensitivity of the test was similar to that of reverse transcription-qPCR assay (60). Thus, some advantages can be proposed for the one-pot assays such as STOP, (i) it is simple and fast (10–15 min for concentration range of femtomolar and less than 1 h for a concentration range of attomolar), (ii) it causes less contamination, (iii) it is easy to obtain quantitative results. However, there is also drawback with the one-pot assay, as it is less sensitive than two-step methods. To address the compromised sensitivity in one-pot strategy, dual crRNAs specific to a single gene could still provide high sensitivity as low as 5 copies as shown by AIOD-CRISPR method (62). One-pot assay is proposed for the applications that are time-sensitive, quantitative, require high-throughput, and carry a significant risk of contamination (e.g. in repeated testing). In contrast, two-step is proposed for applications with challenging sample inputs for example quick extractions from body fluids like saliva or urine (93).

The use of miRNAs as a clinical cancer biomarker is negatively affected by the absence of accurate, fast and cost-effective assays. However, development of one-step and one-pot isothermal assay called Endonucleolytically Exponentiated Rolling Circle Amplification with CRISPR-Cas12a (Extra-CRISPR) offers a rapid, specific and high sensitivity detection method for miRNA. This technique offers several advantages compared to previously reported CRISPR-based biosensing methods including the strategy to harness both *cis-*cleavage and trans-cleavage activities of CRISPR-Cas12a system, building a robust one-step, single tube isothermal assay for miRNA analysis by incorporating multiple reactions into one coupled reaction network. This provides a reasonable analytical performance similar to reverse transcription-qPCR, including high sensitivity, single digit femtomolar detection limit, single-nucleotide specificity and rapid and flexible turnaround. Extra-CRISPR has a potential for use in point of care diagnostics as the method follows a simple workflow and requires no specialized instruments (94).

### Nucleic acids extraction

Besides the one pot strategy that simplifies the detection, the streamlined RNA extraction methods are also useful to reduce the liquid-handling steps. For example, magnetic beads purification combines the lysis and magnetic bead–binding steps and eliminates the ethanol wash and elution steps. The method could decrease the extraction time to only 15 min with less hands-on time (60).

**Figure 10.**
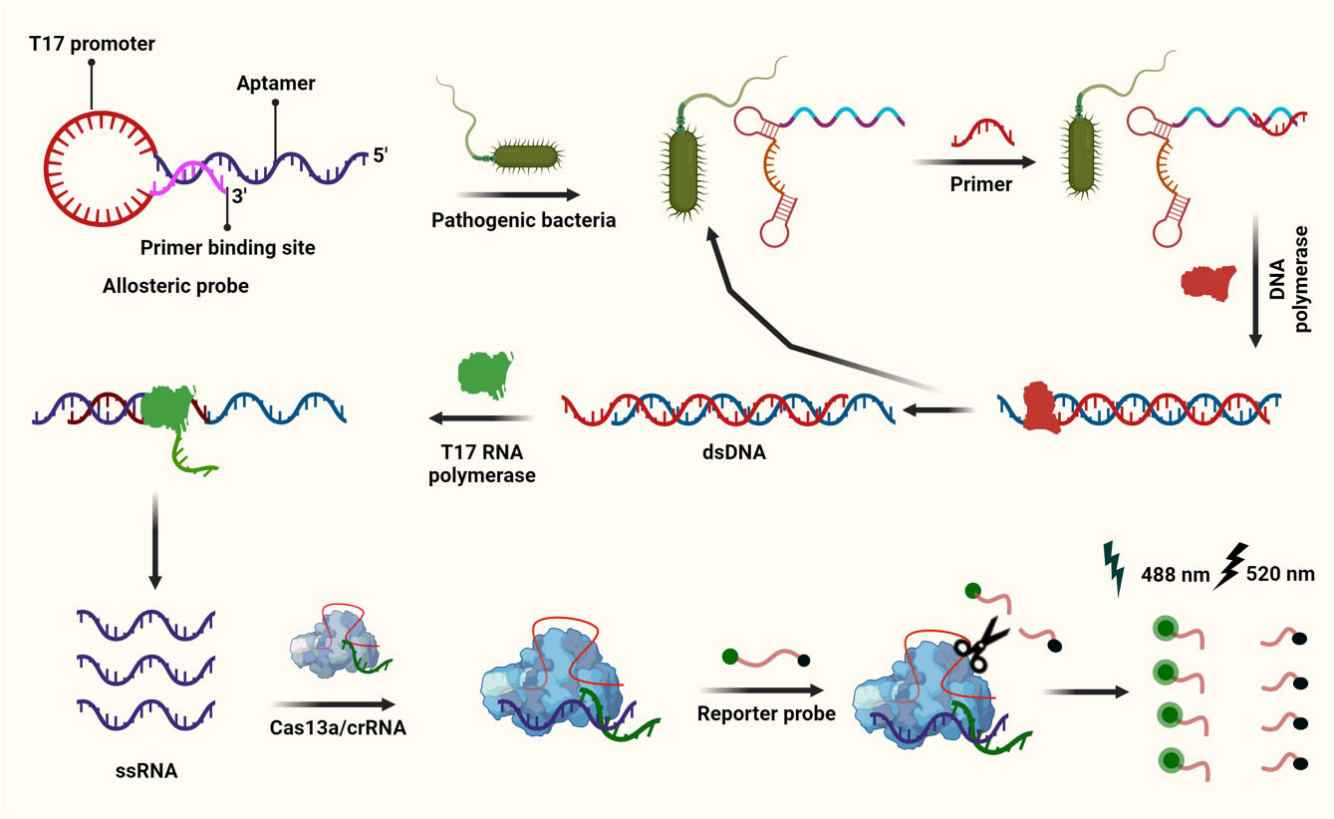
Allosteric probe-initiated catalysis and CRISPR-Cas13 (APC-Cas) method. The probe composed of an aptamer domain for recognition of the target pathogen, a primer binding site domain, and a T7 promoter domain. The aptamer domain binds with the target pathogen. Then, primers annealing to the primer binding site domain yields a dsDNA by addition of DNA polymerase. T7 RNA polymerase identifies T7 promoter sequence on the dsDNA and generates ssRNAs. ssRNAs hybridized to their complementary sequence in designed guide RNA of Cas13-crRNA complex, activates the non-specific collateral cleavage of RNA reporter probe by Cas13, thus producing fluorescence signals (95).

To simplify the detection, one may even consider the nucleic acid extraction free detection methods to minimize the number of liquid handling steps. As an example of DNA extraction free Cas13 detection, Shen *et al.* presented ‘allosteric probe-initiated catalysis and CRISPR-Cas13’ (APC-Cas) method using a specific allosteric probe (Figure 10). The probe is a ssDNA molecule, that is composed of three functional domains: an aptamer domain for recognition of the target pathogen, a primer binding site domain, and a T7 promoter domain. Once target pathogen is present, the aptamer domain of probe binds with the target pathogen. Then, the hairpin structure of the probe is unfolded. This allows primers annealing to the primer binding site domain and yields a dsDNA by addition of DNA polymerase. Consequently, T7 RNA polymerase identifies T7 promoter sequence on the dsDNA and generates ssRNAs by transcription. ssRNAs are then hybridized to their complementary sequence in designed guide RNA of Cas13-crRNA complex. It finally activates the non-specific collateral cleavage of RNA reporter probe by Cas13, thus producing fluorescence signals (95).

In addition, the detection of biomarkers from clinical samples is challenging due to limited purity and integrity. Despite the successful detection of target DNA/RNA from body fluids such as urine by HOLMESv2, the detection limit for target RNA is low, most likely due to degradation of RNA in the urine environment.

### Amplification free methods

Basically, to achieve higher sensitivity by HOLMES detection, it has been argued that the amplification is needed (59). However, Fozouni *et al.* proposed an amplification free Cas13-based detection for sensing SARS-CoV-2 that could detect 100 copies of viral RNA per μl of nasal swab RNA. Multiple crRNAs used for targeting different parts of the viral genome enhance the sensitivity of the test without any amplification. Similarly, Wang *et al.* provided a solution for amplification bias, complicated operation, complex instruments, and aerosol pollution by developing integrated assay for single molecule digital detection of nucleic acids on a CRISPR-Cas13 and microarray. The implemented magnetic beads pool the target away from the untargeted sample. This aids significantly to improving sensitivity and specificity, whilst reducing the detection time. The limit of this assay for amplification-free detection of SARS-CoV-2 is 2aM (96).

**Figure 11.**
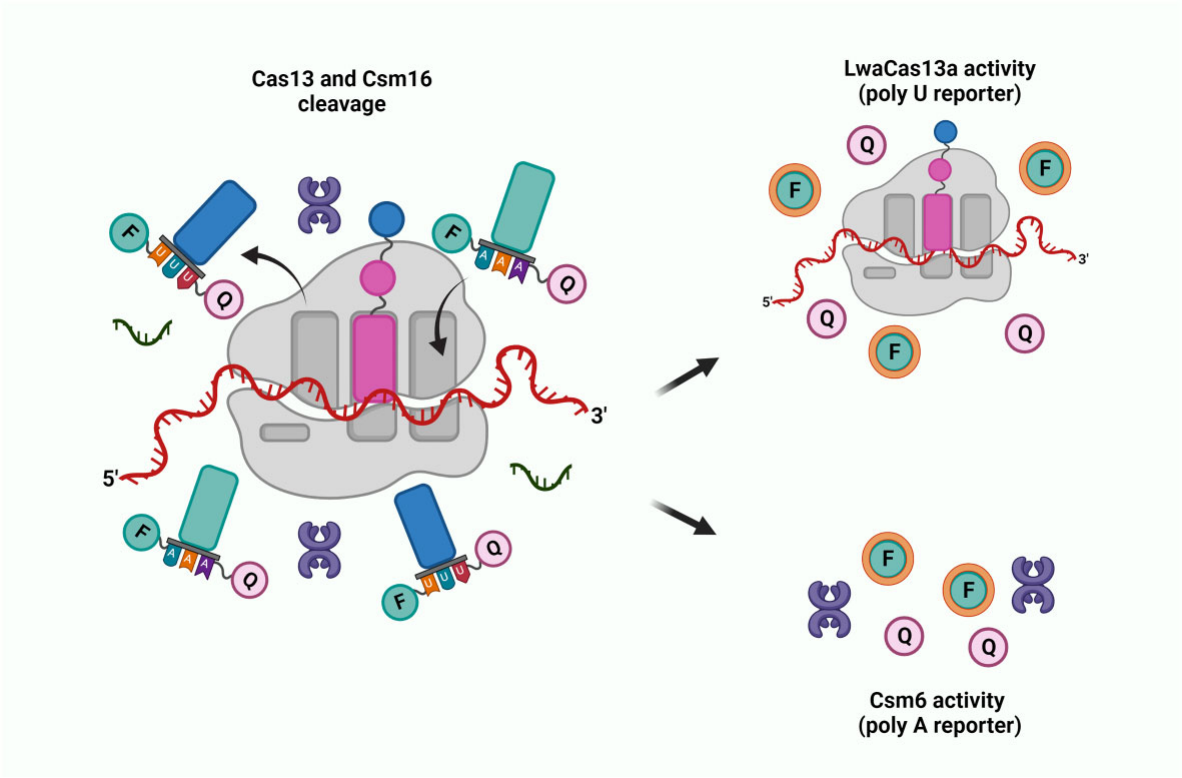
Combined LwaCas13 with CRISPR type III RNA nuclease Csm6 to detect single molecules of RNA or DNA with increased sensitivity and without pre-amplification step (39).

Gootenberg et al., (2018) combined LwaCas13 with CRISPR type III RNA nuclease Csm6 to detect single molecules of RNA or DNA with increased sensitivity and without pre-amplification step (39) (Figure 11). Another study reported a unique platform for highly specific and sensitive detection of *Staphylococcus aureus (S. aureus)* based on Aptamer-based Cas14a1 Biosensor (ACasB) which did not require nucleic acid or amplification processes (35).

In summary, CRISPR-based methods have been shown to detect unamplified nucleic acids with fM level sensitivity. However, their LOD greatly varies depending on the detection methods used. For instance, among most of the detection methods used in CRISPR-Cas systems, electrochemical-based methods are cheap, portable, and allow simple samples processing, but their long detection time is considered disadvantageous. On the other hand, CRISPR-Cas13-based droplet microfluidics technology showed sensitive detection with a LOD of 10 aM. However, the output reading highly depends on coat-ineffective and bulky optical equipment (97).

### PAM sequence and guide sequence

One issue with the detection of double strand target sequences by Cas12 is that the presence of PAM sequence is required for detection. However, one could overcome the PAM sequence limitations by introducing the PAM sequence in the primer for DNA amplification and further detection by Cas12 (17). Zhang *et al.* engineered various nicked dsDNA activators containing PAM nucleotide deletion to demonstrate Cas12a protein had different interaction with the nucleotides in PAM sequence. Particularly nucleotides at the 5′ terminal showed the largest effect on trans-cleavage activity with utmost sensitivity and specificity (98). Though this approach cannot be applied to the amplification free detection systems.

An optimal detection method should be able to discriminate single-base differences. It was already found that the 5′-end seed regions in the crRNA guide sequence is extremely important for Cas12a-based sensing by HOLMES (59,99). Additionally, short guide sequences (1–16 nt) are recommended to be used in HOLMES for sensing single base differences (59).

A robust gRNA requires maximum on-target efficiency and minimum off-target specificity. To achieve this, various computational methods are adapted for high-efficiency CRISPR gRNA design (100). For instance, CHOPCHOP has a clear interface with well-rounded functions, >200 genomes are listed on the webpage where users can input gene names, genomic coordinates, or target sequences and use the function ‘Find Target Sites’. By using this function, the page shows the results in a table where each gRNA has a rank, genomic information, GC content etc. This method is compatible with and can support several experimental procedures including CRISPR Cas9 nuclease/nickase, Cpf1, CasX, Cas13 and TALEN (101,102). Of note, these predictive technologies were designed for individual experiments and rules and therefore each model generates distinct on-target scores for sgRNAs. Therefore, users should be cautious in choosing models that fir their own experiments, and would be advised to use multiple models to evaluate gRNA before reaching a conclusion (100). For instance, one can improve CRISPR guide design by using consensus approaches by combining sgRNAScorer2, CHOPCHOP, PhytoCRISP-Ex and mm10db tools. One can generate a set of guides where up to 91.2% are efficient but still carries some disadvantages such as time consuming, and there are limitations in using these tools to compute resources and scalability (103).

An improved CRISPR-Cas system using 2′-O-methylated gRNA to promote Cas12a protein specificity outperforms existing SNP detection methods by a factor of three for single SNP detection. It indicates that 2′-*O*-methylated gRNA can be used to improve Cas12a's specificity for a variety of applications (104). Another study found that including non-canonical universal bases into Cas9/Cas12a guide RNAs are tolerated and thereby specificity is selectively degenerate at the site of universal base incorporation. This technology could target a series of polymorphic gene variants using a single guide RNA. Moreover, this technology can even be used to avoid false-negative results in diagnostics caused by pathogen evolution (105).

### Type III CRISPR-Cas system

A completely distinct class of CRISPR-Cas systems is the type III effector. A recent study identified a novel type III-E like effector which composed of Cas7 like and a Cas1-like domain, that can be engineered into an active chimeric RNA-targeting Cas effector to bring up a new role of Cas1 in RNA targeting. Although this research reported clear evidence of RNA-targeting *in vitro* and in mammalian cells, they did not outperform WT DiCas7-11, suggesting the need to improve Cas7-S effectors engineering. Apart from this, RNA cleavage by Cas 7–11 enzymes is slow and therefore increasing their catalysis rate to enhance the enzyme activity while maintaining the high target specificity is required (106). In another recent study, type III CRISPR-based TEAR-CoV was applied against viral replication and function, however, their safety and efficacy of TEAR-CoV warrants future study to improve this system for anti-viral treatment (107). Although Type III CRISPR-Cas unique targeting mechanism provided a major breakthrough towards understanding the CRISPR-Cas systems, many questions are still unanswered. There are contradictory results reported on preliminary studies about the presence of RNA, PAM or rPAM, a short sequence motif flanking the target RNA that is crucial for DNA degradation (108), whereas other studies emphasized the complementary between the crRNA tag and the repeat sequence flanking the RNA spacer and protospacer sequences are vital for targeting of CRISPR array (109–111). Moreover, studies have reported a significant degree of tolerance to mutations in the target seed sequence by Type III systems (112–115). Future work is needed to confirm the requirement of PAM and/or seed sequences for Type III immunity and to confirm whether they aid target detection.

### LOD limitations

The clinically used nucleic acids detection assays require lower LOD and preamplification steps. However, most of the CRISPR-based diagnostics have reported LOD in the picomolar range when Cas enzymes are used without upstream preamplification of the target (116,38). This range of LOD requires a relatively high concentration of DNA or RNA in the sample for target detection that is one of the disadvantages when selecting pre-amplification free detection technique. For example, during early phase of infection, there is a high concentration of severe acute respiratory syndrome coronavirus 2 (SARS-CoV-2) (∼6.76 × 10^5^ copies per swab) that is required for pre-amplification-free identification (46). In the Tables 1–3, we present an overview of the sensitivity as LOD per method. Whilst LOD is potentially indicative of sensitivity, this remains controversial as LOD is clearly determined by the pre-amplification step (RPA, LAMP, PCR), which are variable tests and independent of CRISPR Cas enzymes. However, in case of amplification-free Cas12 and Cas13 diagnostics, Huyke *et al.* showed that the enzyme kinetics and detector sensitivity are the two principal factors that determine the LOD of amplification-free systems (48).

**Table 3.** Cas-based detection of nucleic acids in clinical samples

Protein	Detection molecule	Gene	Patients	Detection limit, operation time, cost, point of care (POC), orthologue, temperature (Tm) control, single base resolution	References
Cas13	RNA	169 viruses, subtyping of influenza A strains, HIV drug-resistant mutations	58 plasma, serum, and throat and nasal swabs, 22 samples from patients with HIV	Attomolar, 3 h, low cost with multiplexing and throughput, LwCas13a, Tm control (37°C)	(44)
Cas13a	RNA	MicroRNAs MiR-17, -10b, -155, -21	1–20 tumor tissue samples from 20 breast adenocarcinoma patients	4.5 amol, <30 min, POC, LbuCas13a, constant temperature 37°C, single base resolution	(131)
Cas13	RNA collected from urine, whole blood, plasma, serum, saliva	Zika and Dengue viruses	53 Zika virus patient samples, 24 Dengue virus samples	For Zika virus 45 copy/μl in whole blood and serum, 1 copy/μl in saliva, 10 copy/μl in urine, <2 h, LwCas13a, nuclease inactivation 37–50°C; viral inactivation 64–95°C; Tm control (37°C for fluorescence and RT for lateral flow), single base resolution	(40)
Cas13 with Csm6	Isolated cfDNA from liquid biopsy	*EGFR* (L858R), (T790M), exon 19 deletion (five amino acids)	4 patients	2 amol, <90 min, low cost for many targets detection, LwaCas13a and EiCsm6, Tm control (37°C), single base resolution	(39)
Cas13	RNA	SARS-CoV-2	50 unextracted nasopharyngeal swabs	>1000 copy/μl 50 min, LwCas13a, Tm control (37°C), single step	(42)
Cas12	RNA, gene N encoding nucleocapsid protein	SARS-CoV-2	200 nasopharyngeal swabs	33 copies per milliliter, less than 1 h, AacCas12b and AapCas12b, one pot in a single temperature	(60)
Cas12	DNA collected from plasma	*EGFR* mutation (L858R, T790M)	28 lung cancer patients, 20 cancer-free individuals	0.005%, <3 h, LbCas12a, preamplification and 37°C	(78)
Cas12	RNA extracted from nasal swabs down to	E (envelope) and N (nucleoprotein) genes of SARS-CoV-2	50 nasopharyngeal patient samples	10 copies/μl, <40 min, POC, LbCas12a, Tm control (37°C), single base resolution	(61)
Cas12	DNA collected from anal swabs	HPV16, HPV18	25 patient samples	Attomolar, 1 h, POC, LbCas12a and SpCas9, Tm control (37°C)	(16)
Cas9	DNA	Mutations for Duchenne muscular dystrophy in exon 3 or 51	Buccal swabbing	1.7 fM, 15 min, dCas9 (University of California, Berkeley MacroLab), Tm control (37°C)	(89)
Cas9	DNA, RNA from respiratory fluid and dried blood spots	Antimicrobial resistance genes in pneumonia-causing gram-positive bacteria, drug resistance in the malaria parasite Plasmodium falciparum	9 patient samples	Sub-attomolar, low materials cost, S. pyogenes Cas9	(74)
Cas9	DNA from cervix brush	L1 fragments of HPVs	30 HPV-positive clinical samples	1 ng (SiHa gDNA) and 5 ng (HeLa gDNA), 2–3 h, Cas9 from New England Biolabs, 37°C, 72°C, 95°C, 58°C	(72)

cfDNA, cell-free DNA.

Chen *et al.* and Slaymaker *et al.* are the two seminal papers that set a high bar for enzyme kinetic performance and identified Cas12a and Cas13b as diffusion-limited enzymes with high turnover rates of 1250–1000 s^−1^. Since then, many international researchers in the field corroborated their CRISPR rate measurements and reported even higher kinetic rates by incorporating modifications. In line with this, a recent perspective article (117) debated the limits of LODs as the application of CRISPR-based diagnostics remains an open question due to the reports of kinetic rates that have been largely inconsistent and contains gross errors including violations of basic conversation and kinetic rate laws. This perspective came forward to pinpoint the drawbacks thereby future researchers would need to pay attention and identify strategies to improve kinetic rates and LODs, therefore CRISPR field would benefit from verifications of self-consistency of data and providing sufficient data for experiment reproducibility (117).

### Clinical samples

One of the challenges with the detection systems described in this review, is that they are clinically tested against small sample cohorts (Table 3). However, to properly validate the clinical significance of the CRISPR system for the detection of nucleic acids from clinical samples, sufficiently large sample size and benchmarking against routine methods like PCR and sequencing is needed.

### Quenching ability of nanomaterials used in detection

When it comes to readout, AuNP probes possess exceptional optical properties including larger molar absorption coefficient than small fluorescent dye molecules, as well as localized surface plasmon resonance. Hence, colorimetric readout using AuNP probes can be used for sensitive detection of nucleic acids by Cas12a. The assay can be visualized by naked eyes without need of any sophisticated optical instrument, that makes AuNP-based colorimetric detection cost-effective diagnostics (86,118–120).

The fluorescence quenching in the absence of target nucleic acid occurs due to distance-dependent fluorescence resonance energy transfer (FRET) effect. The quenching ability of different nanomaterials such as spherical AuNPs and 2D graphene oxide nanosheets were compared. It was indicated that graphene oxide possesses better quenching ability and signal recovery than AuNPs (84).

## Essential points for design of ideal Cas-based detection system

Based on this review, we outline the essential points that would need to be considered for designing an ideal Cas-based detection system.

- A nucleic acid extraction free protocol for sample preparation is required to reduce the number of steps and the overall sample-to-answer turnaround time. One-pot strategy with simple nucleic acid extraction method using beads can be recommended.
- Isothermal (single temperature) amplification methods including recombinase polymerase amplification and loop-mediated isothermal amplification are proposed to eliminate the need for thermocyclers and make the technology cost-effective.
- Amplification free detection Cas systems can be developed, however, to ensure sensitivity, multiple crRNAs targeting multiple regions in the target genome can enhance the sensitivity.
- The PAM sequence can be introduced by the primer used in target amplification; thus, PAM limitations can be lifted.
- The sequence and size of crRNA would need to be optimized for sensing the single base difference.
- The ideal detection system is that the steps performed at ambient temperature in less than 15 min and via a colorimetric readout that does not require opening the tubes and can be visualized by naked eyes.
- Formulations for the assay can be lyophilized, simplifying the distribution and assay preparation.
- The detection systems can be advanced by integrating CRISPR assay into a disposable microfluidics chip platform.

## Data Availability

No new data were generated or analyzed in the support of this study.
